# Filtering for truth: high-precision taxonomic classification in nanopore shotgun metagenomics data through a KMA-based bioinformatic pipeline (KAPTAIN)

**DOI:** 10.1186/s12864-026-12668-0

**Published:** 2026-02-24

**Authors:** Alexander Van Uffelen, Andrea Gobbo, Marie-Alice Fraiture, Andrés Posadas, Nancy H. C. Roosens, Kathleen Marchal, Sigrid C. J. De Keersmaecker, Kevin Vanneste

**Affiliations:** 1 Transversal activities in Applied Genomics, Sciensano, Brussels, Belgium; 2 Department of Information Technology, Internet Technology and Data Science Lab (IDLab), Interuniversity Microelectronics Centre (IMEC), Ghent University, Ghent, Belgium; 3 Department of Plant Biotechnology and Bioinformatics, Ghent University, Ghent, Belgium

**Keywords:** oxford nanopore technologies, nanopore sequencing, shotgun metagenomics, taxonomic classification, performance evaluation

## Abstract

**Background:**

Shotgun metagenomics enables to study microbial communities without biases from culturing and isolation, but taxonomic classification to the species level remains challenging due to high false positive rates. Oxford Nanopore Technologies offers new opportunities to address these challenges by producing longer reads. However, different pipelines and tools use different methods to reduce false positives, resulting in variable outcomes with limited exploration of what works best in practice. Relative abundance filtering is often used to improve precision by removing false positives but reduces also recall by removing true positives. In this study, we optimized a broadly applicable taxonomic classification pipeline for long-read nanopore sequencing data that improves precision. The pipeline uses the tool KMA as the underlying classifier, followed by specific post-processing and optimization of filtering thresholds. Based on ten defined mock communities, different filter thresholds were evaluated, alongside the effect of the sequencing yield and the limit of detection (LOD).

**Results:**

Our optimized pipeline substantially outperformed default classifier settings, and the conventionally used relative abundance filtering. Classification accuracy improved with higher sequencing yields, requiring at least a post-filtering yield of 500M bases, and ideally 1000M bases, for reliable results. At yields above 1000M bases, median precision could be improved up to 95% while maintaining median recall at 91.62%. Further increasing median precision to 99% reduced recall to 79.08%. Similarly, higher sequencing yields lowered LOD. For yields above 1000 M bases, the limit of detection remained stable at 0.1% up to a median precision of 95%, while yields below 1000M showed an LOD of 1%. Validation on ten probiotic-derived mock communities confirmed the pipeline’s performance and general applicability.

**Conclusion:**

Our optimized classification pipeline for nanopore sequencing data provides substantially higher precision compared to default approaches and is suitable for diverse metagenomic applications. We provide specific guidance on expected recall and precision values for minimum sequencing yields and their associated LODs. Our optimized pipeline, called KAPTAIN (KMA-bAsed Pipeline for meTAgenomic specIes ideNtification), is publicly available on GitHub (https://github.com/BioinformaticsPlatformWIV-ISP/KAPTAIN) and also the Galaxy instance of our institute (https://galaxy.sciensano.be) to be used by other scientists.

**Supplementary Information:**

The online version contains supplementary material available at 10.1186/s12864-026-12668-0.

## Introduction

With shotgun metagenomics, all genetic content of a sample is sequenced directly, bypassing the need for isolation or cultivation of the microorganisms [1]. This approach can provide a less biased view of microbial communities without requiring a priori knowledge, enabling both high-resolution taxonomic identification and functional analysis [2, 3]. Consequently, its application has found its way into many different domains of life sciences, such as characterizing uncultured microbes in environmental microbiology [4], food safety [5], diagnosing infectious diseases in clinical settings [6], contamination detection in food enzymes [7], and discovering new biosynthetic enzymes in industrial biotechnology [8]. Shotgun metagenomics has become particularly compelling with the advent of long-read sequencing technologies, such as Oxford Nanopore Technologies (ONT). With read lengths of several thousand base pairs (bp), compared to ~ 300 bp for short-read platforms, ONT sequencing can reduce bioinformatics complexity and improve taxonomic resolution [9, 10]. Furthermore, while nanopore sequencing has historically been associated with a lower sequencing quality, ongoing improvements have greatly reduced this issue [11].

Determining the taxonomic composition of shotgun metagenomic data typically involves assigning a taxonomic label to the sequencing reads, which is then used to determine all species present in the sample. Taxonomic labels are assigned by comparing sequencing reads against a reference database using specialized tools called taxonomic classifiers. This comparison is typically done in one of the following ways: through k-mer mapping, which breaks reads into short sequences for rapid exact matching but often without precise reference location; through alignment-based mapping, which compares reads to reference sequences more precisely; or through a hybrid approach combining both methods. Different databases can be used, including whole-genome DNA sequences, protein sequences, or specific marker sequences. Several bioinformatics tools for taxonomic classification have been developed [12], each typically implementing heuristics to reduce the required computational power [13]. Consequently, the large diversity of different approaches and computational tools for shotgun metagenomics has spawned multiple benchmarking studies trying to elucidate the best performing classifiers, both for short-read data [13–15] and increasingly for long-read data [13, 16–18]. Although these studies offered valuable insights into the strengths and weaknesses of different classifiers, they also indicated that there is no definitive classifier. All classifiers typically struggle with low precision, often reporting organisms not present in the samples, and precision can vary widely between classifiers. Recall (or sensitivity) tends to be less problematic and less variable, but many classifiers still fail to detect species present in the samples. Performance is further heavily influenced by the choice of database, which can be biased, incomplete, or contain erroneous entries [19, 20]. Ultimately, these benchmarking studies do not point to a single best-performing approach but instead reveal a diverse landscape with different classifiers performing better for specific application scopes, with even the better performing classifiers exhibiting notable issues.

A major consequence of low precision is the frequent occurrence of false positives [21], i.e., species incorrectly reported as present. As noted above, database limitations contribute to these errors, but other factors are also important. Sequences with high similarity to other species can be misclassified [22], and artifacts produced by the classifier itself can generate additional false positives [13]. Technical contamination, such as DNA from reagents introduced during sample and library preparation (the “kitome”) [23,24], poses an additional challenge. Since these contaminants are not part of the true sample composition, but are unintentionally introduced during wet-lab steps, these species could be considered false positives relative to the study case. Also the sequencing yield can have an effect on the final detected species [25]. In the absence of prior knowledge, as is typical in shotgun metagenomics, addressing these challenges, and consequently eliminating such false positives, remains inherently difficult. Nonetheless, various strategies have been undertaken ranging from software-specific parameters and database optimization [19, 26] to machine-learning approaches [27]. One of the most commonly used methods to remove false positives is, however, simply to set a minimum relative abundance threshold, often based on the number of reads allocated to a certain species, below which the species is considered absent [13]. Selecting an appropriate cutoff that maximizes performance is difficult because microbial communities vary greatly and species present at low abundances can easily be missed. Other metrics can also be used, such as breadth or depth of coverage [28–30].

Our previous benchmarking study identified KMA as one of the better performing classifiers when using default settings for nanopore sequencing data [18]. KMA implements a two-step mapping approach prior to alignment, in which the first step selects a set of target references likely to match and the second step verifies these references [31]. Multimapping query sequences are resolved using the *conclave score* [32]. Although KMA is technically a sequence aligner, we will refer to it here as a taxonomic classifier, since its two-step approach and the use of the conclave score make it a strong-performing method for taxonomic classification. In another recent study, we demonstrated that optimization of KMA by using the metric ‘template identity (ID)’ and additional optimization and filtering steps could substantially improve the default performance [7], by providing an effective threshold for eliminating false positives, while retaining truly present species. However, this finding was optimized for a specific case study, namely the detection of *Bacillus* species in food enzyme samples. The identified taxonomic classification approach using KMA and specific filtering was therefore overfitted to this specific application scope, and cannot be generalized to other shotgun metagenomic datasets. While separate optimizations could, in principle, be carried out for each different application scope, this approach is both labor-intensive and impractical. A more feasible strategy would be to establish a broadly applicable optimization.

In this study, we investigated such a broadly applicable optimized pipeline for taxonomic classification based on KMA. Using ten defined mock communities (DMCs) representing different application domains, including environmental contexts, and the oral and gut microbiome, the taxonomic classifier’s performance was optimized by investigating the effect of template ID thresholds at different sequencing yields. Concurrently, the limit of detection, i.e., the lowest detectable relative abundance, was determined. We confirmed that optimization substantially improved performance, especially in precision, compared to using KMA, or other taxonomic classifiers when run in default mode(s). The optimized pipeline was cast into a user-friendly tool, called KAPTAIN, which automatically selects the optimal template ID thresholds dependent on input settings, such as the sequencing yield. The aforementioned thresholds and their corresponding performances were validated with commercial, independent datasets sequenced in-house, whose composition was known from the provided labels.

## Materials and methods

**Fig. 1:**
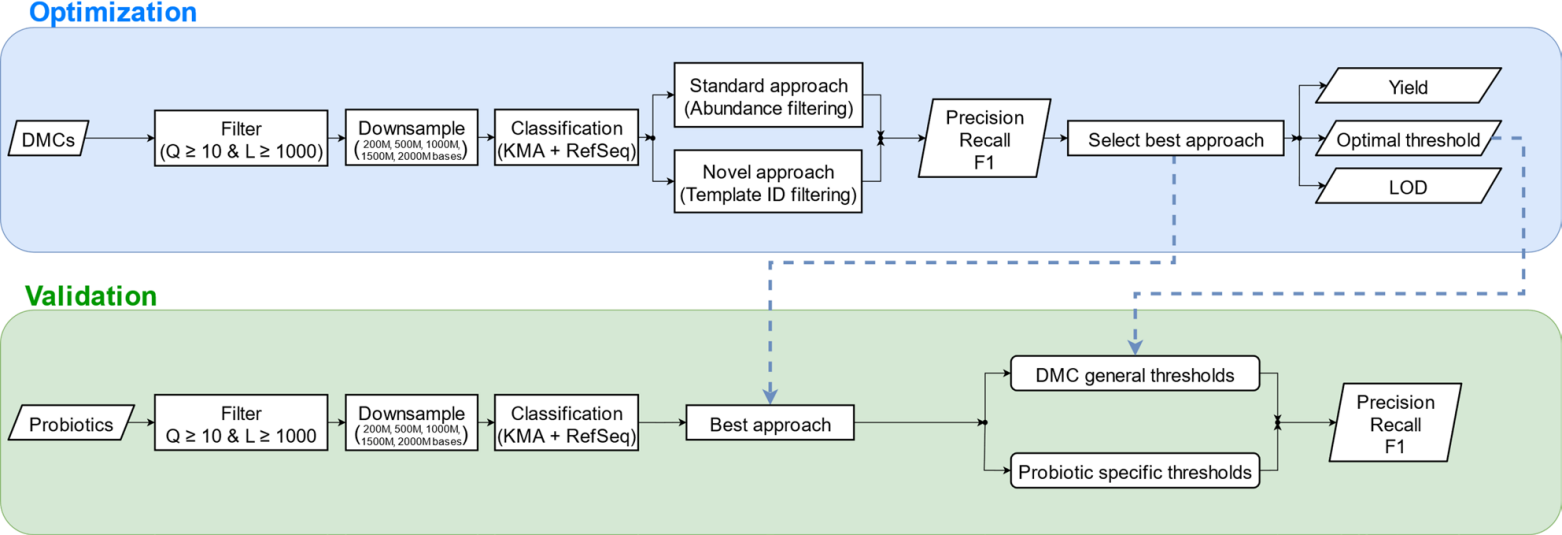
General overview of the optimization and validation phase

An overview of the workflow is shown in Fig. 1. The workflow is divided into two steps: the optimization and validation phase. In the optimization phase, the taxonomic classification pipeline was optimized. In the validation phase, the results of the optimization phase were independently validated.

### Optimization

First, a novel classification approach, integrating both post-processing steps and template ID filtering, was compared to the current commonly used standard approach based on abundance filtering. DMCs, whose composition is known, were used to evaluate performance. Afterwards, the best approach was subjected to a more detailed assessment to determine optimal filter thresholds by investigating the effect of filtering reads below a certain threshold, as well as different yields of the samples and the relative abundance of the present species.

#### Mock communities

Publicly available DMCs were used during the optimization phase to evaluate the performance. A DMC is a laboratory-constructed mixture of known microorganisms with a predetermined composition and often specified abundance levels, which can be used to evaluate analytic workflows by providing a ‘ground truth’. In total, seven DMCs used the R9 chemistry, while three used the R10 chemistry. The samples included a mix of: solely bacteria; bacteria with fungi; bacteria with archaea; or all three groups together. The sources of the DMCs are summarized in Supplementary Table S1 and detailed information on the species names and their sequence abundances can be found in both the Supplementary Text and Supplementary Table S2. To ensure taxonomic consistency between the DMCs and the employed reference database, all species names were updated with TaxonKit 0.18.0 [33] to reflect the taxonomy as of February 2023, when the reference database was created. Additional details on this procedure are provided in the Supplementary Text.

For all but one DMC (HM 277D), abundance information was available. However, not all had the same type of abundance provided. The two most common types of abundance are sequence abundance and taxonomic abundance [34]. Sequence abundance describes the fraction of sequencing reads attributed to each taxon, whereas taxonomic abundance represents the relative number of organisms (or cells) corresponding to each taxon. For consistency, all DMCs not originally expressed as sequence abundance were converted to sequence abundance as described in the Supplementary Text.

As a quality filter, only sequences longer than 1,000 bp and with an average Phred score of at least 10 were retained in the DMCs using SeqKit 2.8.2 [35]. Afterwards, the quality-filtered reads from the DMCs were randomly downsampled to yields of 200M (million), 500M, 1000M, 1500M and 2000M bases, such that each higher-yield subset included the sequences from the lower-yield subset. Sequencing yield affects classification results, and downsampling to varying yields allows determining the point at which results can be considered trustworthy. The downsampling was performed using Rasusa 2.1.0 with the parameter “-s 0”, setting the random seed to 0 to ensure reproducibility [36]. Sequence metrics of the DMCs before and after filtering are summarized in Supplementary Table S3.

#### Classification pipeline

Taxonomic classification of the samples was performed using KMA [32], previously identified as one of the better performing classifiers [18]. KMA version 1.6.2 was used with the following parameters: “-mrs 0.0”, “-bcNano”, “-bc 0.7”, “-ef”, “a”, “-mem_mode”, “-1t1”, “-matrix”, “-tsv” and “-shm”. Afterwards, two approaches were taken to process results: a standard approach based on abundance filtering and a novel approach based on template ID filtering.

The novel approach involves three postprocessing steps after KMA, described in a prior study [7]. In short, the first step removes sequences assigned to plasmid references due to their low discriminatory power (as most plasmids can circulate amongst several host bacteria). Sequences assigned to viral sequences were also removed, as phage hits occur frequently and are often non-specific. Second, reference sequences belonging to the same genome are grouped together and the corresponding metrics are recalculated at the genome level rather than for each individual sequence. Third, for each species, the genome with the highest similarity to the sample, as measured by KMA’s template ID, is selected and other genomes for the species are discarded. The template ID is a metric provided by KMA, calculated based on the number of identical bases between the consensus sequence and the template (i.e., the reference) divided by the template’s length:

1 $$\:Template\:ID=\:\frac{\#Identical\:Bases(Consensus,\:Template)}{Template\:Length}$$

in which the consensus sequence reflects the majority vote of the aligned reads. This way, the template ID is determined by two factors: the breadth of reference coverage and the similarity of the covered part. For example, if a reference of 100 bases is covered across 75 bases, the maximum possible template ID is 75%. This value decreases further if mismatches occur within the covered region, e.g., 5 mismatches within those 75 bases would lower the final template ID to 70%.

The novel approach (i.e., three postprocessing steps and template ID filtering) was compared to the standard approach (i.e., relative abundance filtering). Unlike the novel approach, the standard approach does not include the postprocessing steps from the novel approach, except for the removal of sequences assigned to viral sequences. In the standard approach, the number of aligned reads for each species was summed and then divided by the total amount of aligned reads, resulting in a calculated relative sequence abundance per species. This metric is commonly used both to evaluate classification performance and to filter out incorrectly identified species by applying a minimum abundance threshold [13,17].

For both approaches, a reference database from a previous study was used [7]. Briefly, the database includes assemblies sourced from the NCBI Reference Sequence Database (accessed on 24/02/2023) [37]. For bacteria, only “complete” assemblies were selected; for fungi and protozoa, only “scaffolded” assemblies were used; and for viruses, both “complete” and “scaffolded” assemblies were included. Assemblies containing the term “unclassified” at any taxonomic rank were excluded. Assemblies from the *Bacillus subtilis sensu lato* group were curated based on average nucleotide identity (ANI) and assemblies from the *Bacillus cereus sensu lato* group were replaced with the curated BTyperDB [38]. The resulting database contained 43,456 genomes comprising 3,285,670 sequences. Due to memory requirements of more than 1 TB, the KMA index of the database was built with k-mers prefixed by “TG” by enabling the option “-Sparse TG”, as suggested by the developers^1^.

#### Evaluation

For each yield, evaluation of both the standard and novel approach was done using the performance metrics precision, recall, and F1 score (harmonic mean of the two metrics), based on our previously established evaluation framework [18]. These metrics are calculated based on the number of true positives (TPs), false positives (FPs) and false negatives (FNs). Species that are both present in the ground truth and detected are classified as TPs, while those present in the ground truth but not detected are classified as FNs. Species not present in the ground truth but detected are considered FPs. An optimal classification pipeline detects as many TPs as possible, and by extension no FNs, without introducing FPs.

To reduce the number of FPs, a minimum threshold is often applied where all species falling below the threshold are not considered present. The introduction of a filtering threshold (abundance filtering for the standard approach and template ID filtering for the novel approach) will inevitably also remove some TPs. This trade-off between reducing the number of FPs while retaining a maximum number of TPs was assessed by inspecting the precision and recall at the different thresholds. Precision is defined as the number of TPs divided by the total number of detected species (TPs and FPs). A lower precision means more FPs and/or fewer TPs were detected. The recall (or sensitivity) is defined as the number of TPs divided by the number of expected species (TPs and FNs). A lower recall indicates that fewer TPs are detected. Precision and recall can be jointly summarized with the F1 score. Accordingly, per sample and yield, the precision and recall were calculated continuously across the full range of abundance thresholds for the standard approach or template ID thresholds for the novel approach, from zero to 100%. Then for each yield, the precision, recall, and F1 of all ten samples were aggregated by calculating their medians.

A selection strategy was applied employing either the false discovery rate (FDR) or maximum F1 score to select the best respective abundance or template ID thresholds per yield. The cost of tolerating more FPs is commonly expressed as the FDR, defined as the number of FPs divided by the total number of detected species and equals 100% minus precision. Consequently, an FDR of 5% corresponds to a precision of 95%. More specifically, per yield and FDR, the lowest abundance or template ID threshold was selected that achieved a median precision across samples above the required minimum precision for a target FDR of 15%, 10%, 5% and 1%. Throughout the manuscript, these different FDRs will be referred to as FDR15, FDR10, FDR5, and FDR1, respectively. For the maximum F1 score, a threshold was selected per yield across all samples such that the median F1 across samples was the highest value possible. If multiple thresholds produced the same maximum median F1 score, the one with the highest mean F1 was selected.

The comparison between the standard and novel approaches was conducted using three selection strategies: no filtering, maximum F1, and FDR5. Afterwards, the best approach was then subjected to a more detailed assessment to determine optimal thresholds at different yields and all FDRs. Since abundance information was available for nearly all DMCs, the limit of detection (LOD) was also investigated for each selection strategy and sequencing yield. One sample (HM 277D) did not have detailed abundance information and was therefore not used in the LOD analysis. FNs that occurred because the ground truth species was absent from the reference database were not considered for LOD determination, as these do not reflect the LOD. The LOD was defined as the lowest abundance at which all species with an equal or higher abundance were still detected. To estimate the LOD for each selection strategy and sequencing yield, all nine DMCs were pooled and analyzed collectively rather than calculating LOD on a per-sample basis.

### Validation

Both the standard and novel approaches were also compared using real-life samples, specifically probiotic-derived mock communities (PDMC), as their expected composition is well described. Afterwards, the DMC-derived optimal thresholds from the novel approach were applied to the probiotic samples to validate their performance on an independent test set.

#### Probiotic-derived mock communities

To further evaluate the performance and robustness of the taxonomic classification workflow, ten commercial probiotic products were analyzed as mock communities. These PDMCs were selected based on their declared species composition (labelling of the product) to ensure high overall species diversity across the set, thereby providing a challenging and representative test case for taxonomic classification. Additional details on composition and taxonomy are provided in the Supplementary Text.

Genomic DNA was extracted from the ten PDMC using the Quick-DNA™ HMW MagBead Kit (ZymoResearch), following the protocol described by Van Laere et al. [39], with two modifications : (i) the MetaPolyzyme incubation at 37 °C was extended from 1 h to 5 h, and (ii) the elution volume was increased from 50 µl to 100 µl. DNA concentration was quantified using a Qubit 4 fluorometer (Thermo Fisher Scientific), and purity was assessed with a Nanodrop^®^ 2000 spectrophotometer (Thermo Fisher Scientific) by evaluating the A260/A280 and A260/A230 absorbance ratios. For long-read sequencing, DNA libraries were prepared using the Ligation Sequencing Kit (SQK-LSK114; Oxford Nanopore Technologies), following the manufacturer’s protocol. Each library was loaded onto an individual R10.4.1 MinION flow cell and sequenced for 72 h. Besides nanopore sequencing, the PDMCs were also sequenced with Illumina as an independent sequencing technique, to verify the samples did not contain unexpected species. For short-read sequencing, DNA libraries were prepared using the Nextera XT DNA Library Preparation Kit (Illumina), according to the manufacturer’s instructions. Sequencing was performed on an Illumina MiSeq platform using the V3 chemistry, generating 250 bp paired-end reads.

The Illumina sequences were analyzed using the METACARP pipeline version 0.0.1 [40]. In short, the pipeline first performs a broad taxonomic screening of a sample with Kraken2 [41]. Afterwards, a more detailed mapping is performed with minimap2 [42] against the references found by Kraken2. The METACARP pipeline in combination with the Illumina data was used for two reasons. First, the reference database in this study contained only microbial data. To verify that no plant or animal genetic material was present in the samples, a Kraken2 database that included plant and animal references was used. Second, the pipeline was employed as an independent check to detect any anomalies or unexpected patterns in the samples relative to the provided labels. For this purpose, Illumina sequencing was employed as an independent technology, while all other analyses were performed on the nanopore sequencing data.

For the nanopore sequencing data, the same quality filters, downsampling and both standard and novel approaches were applied, as described in Sections 'Mock communities' and 'Classification pipeline'. Sequence statistics before and after filtering are provided in Supplementary Table S4. The standard and novel approaches were compared, as described in Section 'Evaluation'. Afterwards, for the novel approach, the template ID thresholds obtained from the DMCs across all yields and selection strategies were applied to the PDMCs to assess their impact on performance, using the PDMCs as independent datasets. In addition, PDMC-specific template ID thresholds were established to assess the potential for further application-specific optimization.

For the PDMCs, no consistent information was available on the abundances of the present species. Therefore, no LOD analysis was performed for the PDMCs.

### Availability

The novel approach, together with the optimized template ID thresholds, is available as an open-source command-line tool on GitHub (https://github.com/BioinformaticsPlatformWIV-ISP/KAPTAIN). For non-expert users, the workflow is also accessible through our institute’s web-based Galaxy instance (https://galaxy.sciensano.be) [43]. The database used in this study is available on Zenodo (doi: 10.5281/zenodo.17435876).

## Results

### Optimization of a broadly applicable taxonomic classification workflow using DMCs

During the optimization phase, the novel approach was first compared to the standard approach. Subsequently, the best approach was evaluated in more detail and template ID thresholds were optimized in function of sequencing yield, followed by an evaluation of the LOD.

#### Evaluation of the standard versus the novel approach

**Fig. 2:**
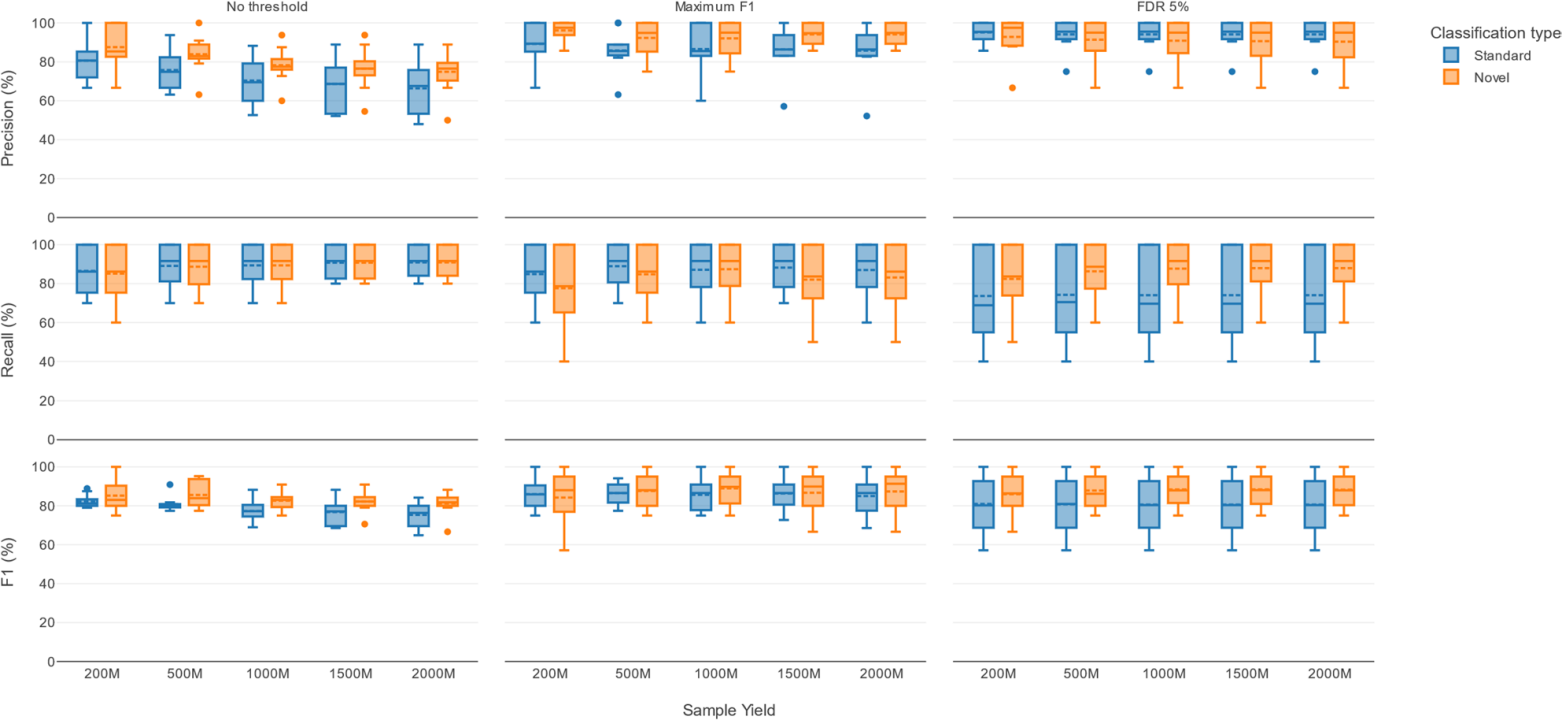
Precision, recall, and F1 scores of the standard and novel approaches applied to the DMCs. Rows show precision, recall, and F1 scores, while columns represent the three selection strategies: no thresholds applied, maximum F1 score, and FRD5. The x-axis shows the five sample yields, whereas the y-axis shows the respective metric values of the ten DMCs expressed as boxplots. Blue and orange boxplots indicate the performance for the standard approach and novel approach, respectively, with solid and dotted lines indicating the median and mean of each boxplot. The corresponding employed filter thresholds are shown in Table S6

Figure 2 shows the comparison between the standard approach (abundance filtering) and novel approach (template ID filtering) in terms of the median precision, recall, and F1 scores, evaluated across ten DMCs using three different selection strategies (no thresholds applied, maximum F1 score, and FDR5) and five different sample yields. The associated number of TPs, FPs and FNs for each combination shown in Fig. 2, are presented in Supplementary Figure S1.

Without any filtering applied, an increasing yield resulted in lower precision for both approaches, but the novel approach consistently achieved a higher median precision across all yields compared to the standard approach. The difference became more pronounced as the yield increased. The main reason for a higher precision of the novel approach was a lower number of FPs (Supplementary Figure S1). In contrast, the median recall of both approaches was identical, since the same median level of TPs were found with both approaches. Consequently, the F1 scores of the novel versus the standard approach were higher across all yields with a median F1 score of 83.03% versus 81.18% at 200M, and 81.75% versus 76.36% at 2000M, respectively.

Using a maximum F1 score selection strategy, the median precision of both approaches increased compared to the unfiltered data. The novel approach was however able to consistently remove more FPs (Supplementary Figure S1), as reflected by higher median precisions across all yields. However, the novel approach also produced more FNs across all yields, resulting in a lower median recall compared to the standard approach. Notwithstanding, the novel approach achieved higher F1 scores compared to the standard approach by a stronger effect of reducing FPs than introducing FNs, with a median F1 score of 88.02% versus 85.87% at 200M, and 91.32% versus 86.56% at 2000M, respectively.

Lastly, using FDR5 as selection strategy, the median precision was consequently close to 95% for both approaches across all yields. The novel approach showed a substantial advantage over the standard approach in median recall. This led for the novel compared to standard approach to higher median F1 scores of 86.39% versus 80.23% at 200M, and 87.95% versus 79.37%, respectively.

In summary, the higher F1 scores of the novel approach across all yields and selection strategies confirmed that the extra filtering steps resulted in a substantial performance boost of template ID filtering compared to abundance filtering. Further optimization therefore focused on the novel approach.

#### Optimization of the novel approach by evaluating the effect of sequencing yield and template ID thresholds

**Fig. 3:**
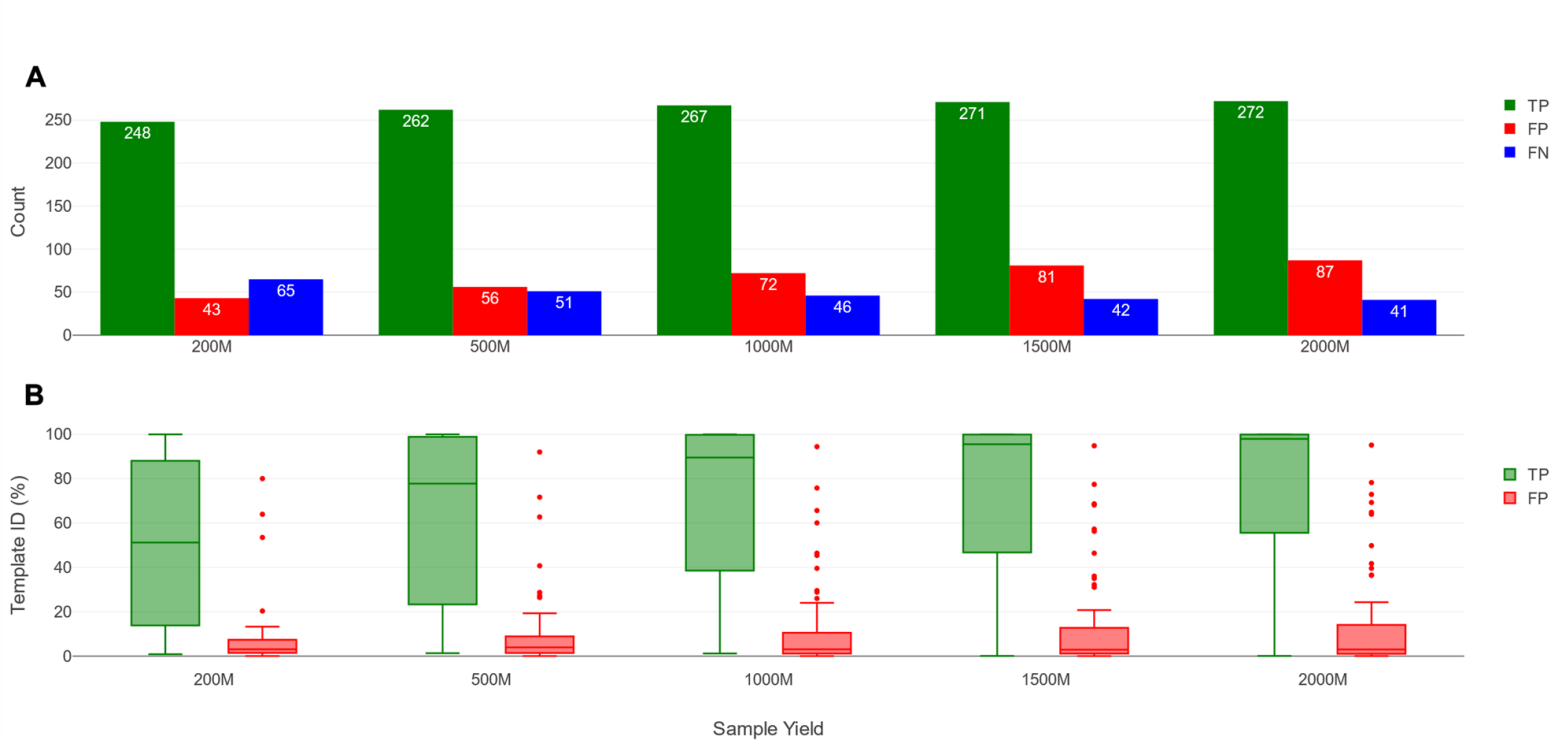
Effects of DMCs’ sequencing yield on TPs, FPs, and FNs for the novel approach **A** Number of TPs, FPs, and FNs for all ten DMC samples using the novel approach without filtering. The x-axis shows the five different yields, while the y-axis shows the counts of TPs (green bars), FPs (red bars), and FNs (blue bars) **B** Template IDs of TPs and FPs for all ten DMCs using the novel approach without filtering. The x-axis shows the five different yields, while the y-axis shows the template ID. Green and red boxplots indicate TPs and FPs, respectively, with a solid line indicating the median. FNs are not shown as they all had a template ID of 0%. For both subplots, no filter thresholds were applied

Next, we investigated in more detail the effects of sequencing yield, and template ID thresholds, for the novel approach. Figure 3A presents the total number of TPs, FPs and FNs across all ten DMCs for each yield, while Fig. 3B shows the distribution of template IDs for the detected TPs and FPs (without any template ID filtering applied). Increasing yields had a positive effect on detecting TPs: the number of TPs increased from 248 at 200M to 272 at 200M out of a total of 313. Conversely, the number of FNs decreased from 65 at 200M to 41 at 2000M. The template ID of TPs also increased, with median values reaching 51.23% at 200M and 97.96% and 2000M. The higher sequencing yields however also resulted in increasing FPs from 43 at 200M to 87 at 2000M. Notwithstanding, despite an increase in the upper range of the FP distribution, the median template ID of the FPs remained relatively stable across yields: 3.06% at 200M versus 2.98% at 2000M. Consequently, increasing yields had a positive effect on the total number of TPs recovered and their template identities, whereas the number of FPs also increased, but their template identities remained largely constant.

**Fig. 4:**
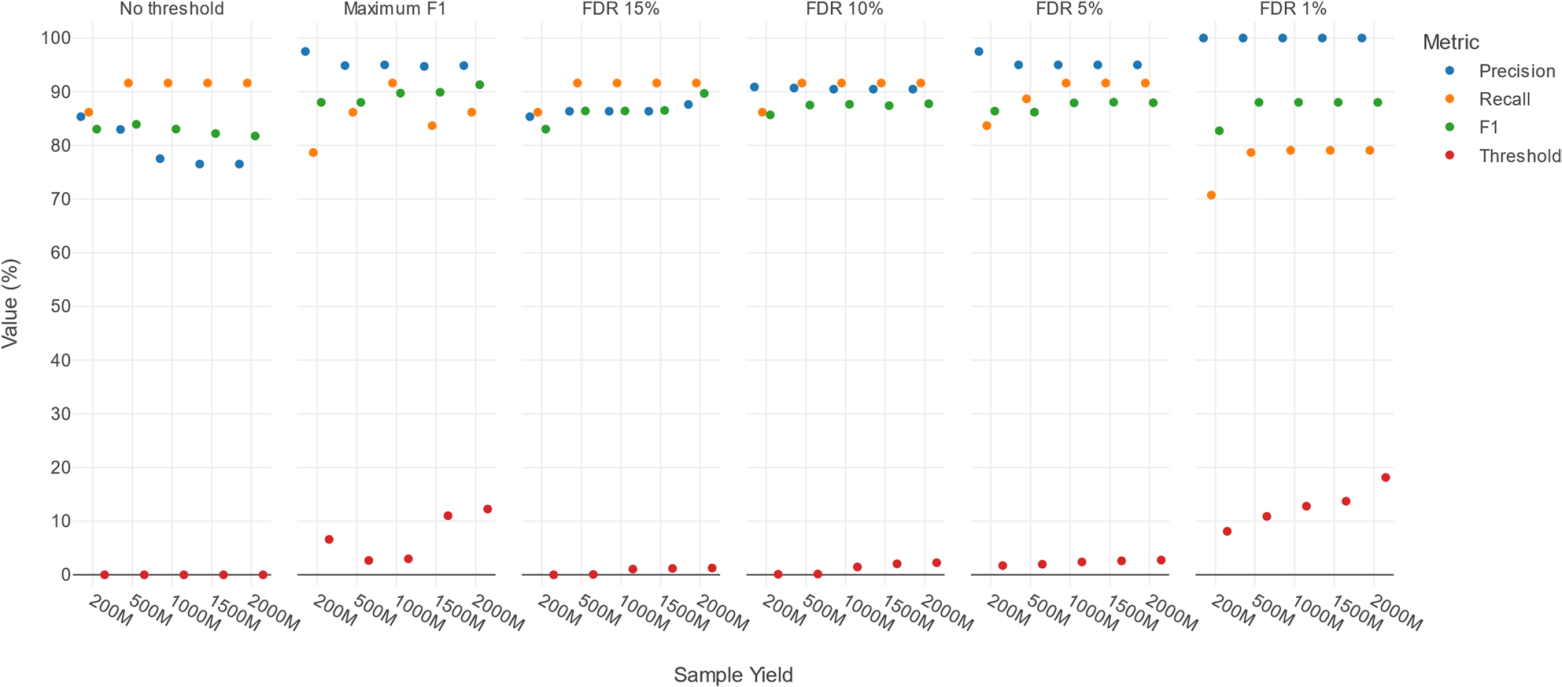
Effects of template ID filtering across different selection strategies and sequencing yields in the DMCs. Columns represent the six different selection strategies. The x-axis represents the different sequencing yields, while the y-axis shows the value (%) of the median performance metrics and associated template ID thresholds. Blue, orange, green and red markers indicate the median precision, recall, F1, and template ID threshold. Details on the applied filter thresholds are available in Table S6 under novel approach

The difference between template IDs of TPs and FPs could hence be leveraged by applying template ID threshold filters to increase classification performance, which was evaluated using six selection strategies: no filtering (to provide a baseline), maximum F1 score filtering, and FDR15, FDR10, FDR5 and FDR1. Figure 4 presents the median precision, recall, and F1 values based on the ten DMCs with the corresponding template ID thresholds (see Supplementary Table S5). Using a maximum F1 score selection strategy, the required template ID per yield was variable with values ranging from 2.67% to 12.25% at the different increasing yields. Decreasing FDR15 to FDR1 required generally higher template ID thresholds. Within each FDR level, a higher yield similarly required higher template ID thresholds, reflecting a slight increase in the template ID values of FPs, especially of some outliers (Fig. 3B). As expected, the median precision largely reflected their respective FDR levels (e.g., median precision of 95% at FDR5 for all sequencing yields), some minor variation notwithstanding. In contrast, the median recall was mostly unaffected by decreasing FDR, with only noticeable decreases at FDR1, and, for lower yields, at FDR5. Higher yields allowed to obtain higher recall at lower FDR levels. This was similarly reflected in the median F1 scores resulting in slightly higher median F1 scores per decreasing FDR setting from FDR15 to FDR1, and showing large F1 improvements going from lower sequencing yields (≤ 500M) to higher sequencing yields (≥ 1000M) across all FDR levels.

In conclusion, without any template ID filtering, higher yields resulted in decreasing median precision due to a strong effect of including many extra FPs detected in the additional reads. In contrast, while higher yields led to more TPs, this translated only into an increase in median recall between 200M and 500M, with no further improvements at higher yields. By applying appropriate template ID filtering, however, the median precision could be increased substantially while having a limited effect on the median recall up until FDR5.

#### Evaluation of the limit of detection for the optimized approach

**Fig. 5:**
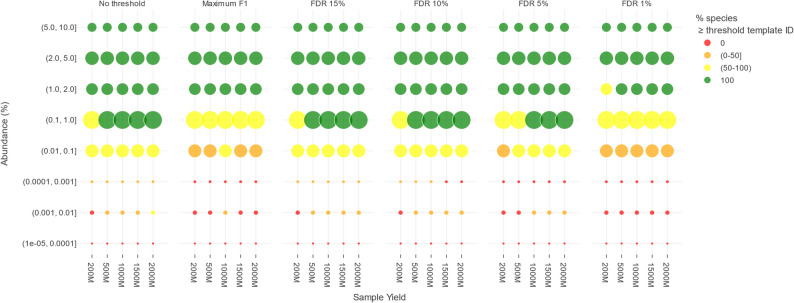
Effects of template ID filtering on detection limits across selection strategies and yields in DMCs. Columns represent the six different selection strategies. The x-axis shows the five sequencing yields, while the y-axis represents the theoretical relative abundance intervals of the ground truth species in the DMCs. Marker colors represent the proportion of expected species meeting or exceeding the template ID threshold at a given relative abundance threshold: green in case all expected species are detected, red if none of the expected species are detected, orange in case more than 0% and up to 50% of expected species were detected, and yellow in case between 50% and 100% of expected species were detected. The size of the marker reflects the number of species. Note that for LOD determination, FNs due to absent species in the database were not taken into consideration

Although the effects of template ID filtering on recall were limited, some TPs were removed. For abundance filtering, it is well described these are typically TPs present at low relative abundances in the samples for which only few reads are sequenced, and we hence hypothesized the same effect was at play when using template ID filtering for the novel approach. Figure 5 shows the number and theoretical relative abundances of the species in the DMCs that remained detectable after applying the associated template ID filtering thresholds depicted in Fig. 4 across different selection strategies and sequencing yields. Note that species not detected because they were absent in the database were not considered for this particular LOD evaluation.

Without applying template ID filtering, the LOD was 1% and 0.1% at yields of 200M and ≥ 500M, i.e., all species present at 1% could be detected at 200M and all species present at 0.1% could be detected at 500M. Using a maximum F1 score strategy, the LOD increased to 1% for all yields. For FDR15 and FDR10, the LOD was the same as without using any filtering. However, for FDR5, the LOD for a yield of 500M increased from 0.1% to 1%. For FDR1, the LOD for yields 1000M to 2000M also increased from 0.1% to 1%, and for a yield of 200M the LOD even shifted to 2%.

In conclusion, template ID filtering could indeed negatively impact the LOD by filtering out some samples present at low relative abundances. This effect was most pronounced when trying to obtain a maximum F1 score or using FDR1, whereas it was absent when using FDR15 or FDR10. At FDR5, there was a limited effect which could be offset by using higher sequencing yields.

### Validation of the taxonomic classification pipeline using PDMCs

Newly sequenced PDMCs were used to independently validate the optimized taxonomic classification workflow. These samples contained defined sets of species according to their product labels and were therefore suitable to serve as mock communities for performance validation. The goal was to assess whether the optimized approach achieved comparable performance on an independent, compositionally diverse dataset.

The Illumina sequencing data from the PDMCs were first processed using the MetaCARP pipeline as an independent control to verify the labelled composition of the samples. Since no inconsistencies were detected in MetaCARP’s results (Supplementary Figure S2), the corresponding nanopore sequencing data were subsequently analyzed using both the standard and novel approaches to evaluate whether the novel approach provided performance improvements as observed for the DMCs.

Afterwards, template ID thresholds as previously optimized using the DMCs were applied to the PDMCs using the same selection strategies. In addition, template ID thresholds were also directly optimized based on the PDMCs themselves and their performance was compared with that of the general thresholds derived from the DMCs.

#### Validation of the standard versus the novel approach using PDMCs

Similar to the DMCs in Section 'Evaluation of the standard versus the novel approach', both the standard and novel approach were evaluated on ten PDMCs using three selection strategies (no thresholds applied, maximum F1 score, and FDR5) and five sample yields. The novel approach outperformed the standard approach on the PDMCs, especially in precision, confirming the results observed for the DMCs that the novel approach performs better. The results are shown in Figure S3 and are further described in the Supplementary Text.

#### Validation of the optimized pipeline using PDMCs

We investigated in more detail the effects of sequencing yields and template IDs for the best-performing approach, i.e., the novel approach, on the PDMCs. In contrast to the DMCs, without filtering thresholds, increasing yields did not result in more TPs, as all 47 TPs were already detected at the lowest yield of 200M (Supplementary Figure S4). However, similar to the DMCs, higher yields increased the median template ID of the TPs. Although generally fewer FPs were observed compared to the DMCs, their numbers likewise increased with yield from 11 FPs at 200M to 36 FPs at 2000M, while the template ID showed a slight decrease. As with the DMCs, these findings indicated that classification performance for the PDMCs could be improved by applying appropriate template ID threshold filters.

**Fig. 6:**
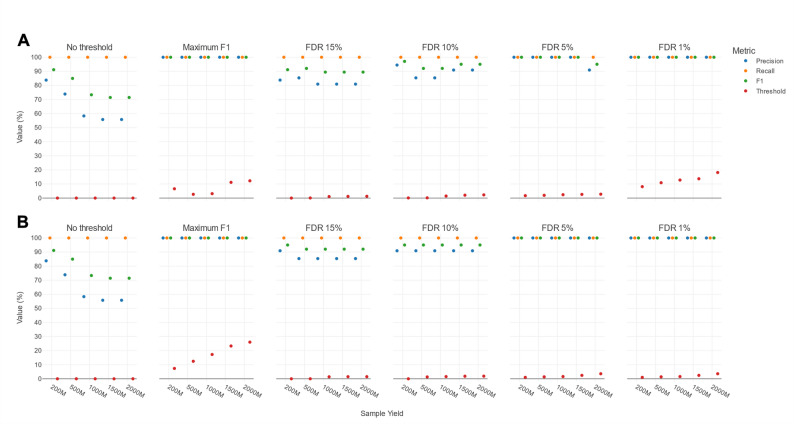
Effects of template ID filtering across different selection strategies and sequencing yields in the PDMCs **A** Using template ID thresholds derived from optimization of the taxonomic classification workflow using the ten DMCs **B** Using template ID thresholds optimized specifically for the ten probiotic samples (tailored specifically to this use case). Columns represent the six different selection strategies. The x-axis represents the different sequencing yields, while the y-axis shows the value (%) of the median performance metrics and associated template ID thresholds derived from the optimization of the novel approach on the DMCs (see also Fig. 4). Blue, orange, green and red markers indicate the median precision, recall, F1 and template ID threshold. Details on the applied filter thresholds are available in Table S6 under novel approach for subplot A and in Table S7 for subplot B

We hence investigated performance using the obtained general template ID thresholds from the DMCs (i.e., thresholds used in our final pipeline) by applying these to the PDMCs. Figure 6A presents the median precision, recall, and F1 values based on the ten PDMCs for the same six different selection strategies (no filtering, maximum F1 score filtering, FDR15, FDR10, FDR5, and FDR1). Without filtering, the PDMCs showed a larger decline in median precision with increasing yields compared to the DMCs (Fig. 4). While larger yields introduced less FPs for the PDMCs versus the DMCs (Fig. 3, Supplementary Figure S4), the negative impact on the PDMCs’ precision was greater. This is because the PDMCs contained considerably fewer species overall, providing fewer potential TPs to counteract the increase of FPs. Additionally, no new TPs were identified at higher yields. In contrast, the median recall of the PDMCs of 100% was higher than for the DMCs. Consequently, compared to the DMCs, without filtering, the novel approach for the PDMCs achieved higher median F1 scores at lower sequencing yields (≤ 500M) but lower median F1 scores at higher yields (≥ 1000M).

Using the general DMC-derived thresholds for the maximum F1 score selection strategy, median precision, recall, and F1 scores of 100% were obtained across all yields for the PDMCs. Consequently, performance was perfect and much higher compared to the DMCs. Using the DMC-derived thresholds for FDR15 and FDR10, a median precision of 85% and 90% is expected, respectively. In practice, the median precisions were a bit lower and varying more across the yields At FDR5 however, the median of all three metrics reached 100%, expect for a yield of 2000M, which only reached a median precision of 90.91%. At the FDR1, all yields reached 100% for all three median metrics. The fluctuation of median precision across FDR settings was caused by the strong influence that a small number of FPs can have on precision (see previous paragraph). Across all selection strategies that included template ID filtering, the median recall was always consistently 100%. Consequently, median F1 scores were much higher than for the DMCs across all yields and selection strategies that included template ID filtering, with as only exception the median F1 score at FDR15 for a yield of 2000M that was marginally (0.25%) lower for the PDMCs than DMCs. Generally, the F1 scores were also more variable due to the larger fluctuation of median precision per selection strategy.

In summary, when the taxonomic classification workflow optimized using the DMCs was applied to the PDMCs, it achieved overall similar or, in most cases, higher performance compared to the results obtained with the DMCs. Moreover, it also led to a notable performance improvement for the PDMCs when compared to results obtained without any filtering.

#### Specific optimization of the novel approach applied to the use case of PDMCs

Fluctuations in median precision and F1 scores among the FDR selection strategies indicated that further optimization tailored to the PDMCs could potentially enhance performance. Therefore, instead of solely adopting the generally applicable DMC-derived template ID thresholds, we also independently optimized these thresholds using a similar strategy as employed during the optimization of the workflow. Figure 6B presents the median precision, recall and F1 values based on the ten PDMCs thereof.

The most notable differences were the thresholds used to reach a maximum F1 score. Whereas the respective DMC-derived template ID thresholds resulted in median values of 100% for precision, recall, and F1 (Fig. 6A), not every individual sample achieved a perfect score of 100% (Supplementary Figure S5). In contrast, the template ID thresholds optimized on the PDMCs were generally higher and resulted in 100% precision, recall, and F1 scores for all samples. This required stricter template ID thresholds of 7.36% at 200M to 26.02% at 2000M compared to the DMC-derived thresholds of 6.60% at 200M to 12.25% at 2000M. For the FDR selection strategies, changes in the template ID thresholds were less profound but still differed, especially for the stricter FDR1, and generally resulted in better and more consistent scores. For instance, the median precisions per FDR selection strategy always reached their respective targets and fluctuated much less than when using the general DMC-derived thresholds. Consequently, F1 scores were generally higher, also at a per-sample level.

In conclusion, for FDR15, FDR10, and FDR5, the general DMC-derived template ID thresholds and their resulting performance were close to those optimized for the PDMCs, although the PDMC-specific thresholds consistently resulted in better performance, particularly with small increases in median precision towards the required FDR setting. The same trend was observed for FDR1 and the maximum F1, although PDMC-specific thresholds differed more noticeably from the DMC-derived thresholds in these cases.

## Discussion

In this study, we optimized and validated a taxonomic classification pipeline for nanopore sequencing data that can be applied on a wide range of metagenomic samples (Fig. 1), demonstrating substantially improved performance compared to running different taxonomic classifiers in default mode(s), particularly in terms of precision. This pipeline is built around the taxonomic classifier KMA, previously demonstrated to be among the better performing classifiers for nanopore sequencing data [18], and incorporates two major enhancements. Firstly, it performs extensive post-filtering of results, including the removal of plasmid hits and grouping of multiple templates representing the same genome in the underlying reference database followed by selection of the highest scoring genome. Secondly, it applies extensive post-filtering of all detected species based on their template ID to differentiate TPs from FPs. The pipeline was optimized on ten DMCs representing different use cases and application domains, and validated on ten PDMCs as an independent test set. Given that sequencing yield can strongly affect taxonomic classification [25], we systematically evaluated the effect of different sequencing yields commonly observed and used in metagenomics studies.

The current state-of-the-art standard approach typically consists of running a taxonomic classifier followed by abundance filtering to discriminate TPs from FPs [13]. The first major difference of our novel approach is the initial post-filtering that specifically removes plasmid hits. Plasmids are mobile elements [44] that typically cross species boundaries, undermining taxonomic specificity. Consequently, even without applying either relative abundance or template ID filtering, the standard approach produced much more FPs across all sequencing yields (Fig. 2). Interestingly, at lower sequencing yields (200M-500M), the standard approach yielded slightly more TPs than the novel approach. This is likely because plasmids are frequently present at high copy numbers, and removing them in the novel approach marginally reduces the detection of some species when read depth is limited (Supplementary Figure S1). However, the median recall remained comparable between approaches, and the effect disappeared at higher sequencing yields.

A second major difference of the novel approach is the use of the template ID for post-filtering. Template ID is a compound metric produced by KMA that combines template coverage and ID, in contrast to relative abundance filtering in the standard approach. Both types of filtering were evaluated using two selection criteria (the maximum F1 score and an FDR5), established by setting thresholds for either relative abundance or template ID. For both, the novel approach consistently scored higher median F1 scores than for the standard approach (Fig. 2). Unlike (relative) abundance, which only incorporates prevalence information, template ID directly measures correctness and completeness, giving more discriminatory power to separate TPs with higher template ID from FPs with lower template ID (Fig. 3). However, perfect separation between TPs and FPs was not achievable due to some overlap between TPs with low template ID and FPs with high template ID. TPs with low template ID were generally caused by the presence of species with low abundances in the DMCs (Fig. 5, Supplementary Figure S6). For low-abundance species, there are not enough reads to cover the reference genome completely. This effect was especially pronounced for species with larger genomes, such as *Cryptococcus neoformans* (18.9 Mb), *Saccharomyces cerevisiae* (12.1 Mb) and *Candida albicans* (14.1 Mb), which required a higher yield to achieve comparable template ID values to smaller genomes with similar abundances.

FPs with high template ID could be primarily attributed to three factors: erroneous reference annotations in either the ground truth or database, the presence of a species in the ground truth for which multiple very closely related species were falsely detected, or the absence of the correct reference species from the database. An example of database misannotation was observed in sample HM 277D, where a high-scoring FP was identified as *Schaalia odontolytica*. In the ground truth, the newer annotation *S. dentiphila* was present, which represents an updated annotation. While NCBI improves their taxonomic assignment by ANI values of assemblies against type strains, no database is error-free nor complete [45], and the taxonomy of certain bacterial groups is still shifting rapidly to which databases cannot always keep up [46]. An example of ground truth misannotation was observed in the strain madness samples, where the listed species *Nostoc sp. PCC 7120 = FACHB-418*, an unclassified member of the genus *Nostoc* on NCBI, was classified as the FP *Trichormus variabilis*, despite the presence of seven other *Nostoc* species in the database. This suggests the genome is likely misannotated in the ground truth, a conclusion supported by independently running GTDB-tk on its genome assembly, which assigned the genome to the genus *Trichormus* (unpublished results) [47]. An example of multiple closely related species was often observed for the genera *Streptococcus*, *Bacillus* and *Thermotoga*. When such species were present in the DMCs, they were typically correctly identified as TPs but associated with multiple FPs with high template ID belonging to the same genus. It is widely recognized that bacterial taxonomy is riddled with phylogenetic inconsistencies [48] and uneven application of ranks, where heavily studied lineages are split into more taxa than equally deep but less explored branches [49]. Consequently, the ANI between different species within a genus can range from 62% to 100% [50]. Species of the genera *Streptococcus*, *Bacillus* and *Thermotoga* have been reported to be closely related and therefore face ongoing taxonomic challenges in the age of genomics, complicating taxonomic classification, in contrast to less closely related species [51–53]. Notably, the more species are sampled and added to reference databases over time, the less straightforward species boundaries will become [20]. Lastly, an example of the absence of the correct reference species from the database was observed for *Pseudomonas marginalis*, giving rise to multiple FPs from the genus *Pseudomonas*. More comprehensive and higher-quality reference databases can help reduce FPs to some extent, but they will not completely eliminate them.

Although recall was generally high to very high, some FNs remained, primarily due to two factors: absence from the reference database and low abundance. Species present in the sample but not represented in the database will inevitably be undetected (and may instead be misassigned as a FP as mentioned above). Examples of species present in the ground truth but absent from the reference database included *Herpetosiphon aurantiacus*, *Pseudodesulfovibrio mercurii* and *Methanomassiliicoccus luminyensis*. Low-abundance species are more vulnerable to technical biases and losses during sample preparation. For example, they may be underrepresented due to inefficiencies in DNA extraction or library preparation biases [30], or simply fail to reach sufficient sequencing depth for reliable detection [25]. As a result, low-abundance species have a higher chance of not being detected, especially if any type of filtering on either abundance or template ID is applied afterwards. For instance, *Enterococcus faecalis* and *Staphylococcus aureus* present at 0.001% and 0.000089%, respectively, were not detected. These observations indicate that recall in real samples can be limited due to insufficient reference database representation and sequencing depth. While the workflow maximizes precision and maintains high recall, very low-abundance species or taxa missing from the database remain difficult to detect, highlighting the potential benefit of deeper sequencing or expanded reference databases.

Sequencing yield affected performance and required adapted template ID thresholds (Fig. 4). Higher sequencing yields enabled the recovery of more TP species and hence higher recalls. Without any template ID filtering however, the increase of TPs was outweighed by the influx of FPs, with as only exception the jump from 200M to 500M, where the growth in TPs outweighed the increase in FPs. The number of additional TPs saturates at higher yields, while the number of FPs can keep increasing. When template ID filtering thresholds were applied, however, higher yields tended to produce higher F1 values because the influx of TPs was not anymore outweighed by the influx of FPs. The results indicated that at least a yield of 500M, and ideally 1000M, is desirable. Yields beyond 1000M still provided additional benefits, but were less pronounced. Notably, different sequencing yields required different template ID thresholds, often being stricter at higher yields, highlighting the importance of adapting thresholds based on the achieved sequencing output.

The employed selection strategy also had an effect on performance (Fig. 4). As expected, selecting the maximum median F1 scores yielded the highest median F1 scores but this approach exhibited considerable variability across samples. As F1 is the harmonic mean of precision and recall, high F1 scores as selection strategy can result in more unpredictable contributions of either the precision and/or recall to this F1 score. In contrast, using a fixed FDR, although not necessarily providing the highest F1 scores, provides a predictable and easily interpretable assessment of expected precision and recall. The choice of FDR setting depends on the use case and application domain. If reducing FPs is important while maintaining recall, FDR5 is appropriate, providing a median precision of ~ 95%, and median recall of 91.62% when the sequencing yield is at least 1000M. FDR1 offers even higher precision, but at a cost of a notable recall reduction. When sequencing yield is lower than 1000M, FDR10 is preferred. If retaining nearly all TPs is more important, FDR15 is recommended. Finally, for applications requiring the highest possible recall, no filtering should be applied, though this substantially reduces precision.

Template ID is calculated based on both template coverage and sequence similarity, which each contribute differently to the final filtering thresholds, as detailed in Table S5. The sequence similarity typically fluctuated between ~ 85% and 95% across different selection strategies. Higher similarity requirements are expected, as KMA and by extension the pipeline, are designed to identify species present in the database rather than performing novel species discovery. However, similarity was never close to 100%, indicating that exact matches are not required for detection. This suggests that the pipeline tolerates a degree of divergence between sample sequences and the database references. Stricter selection strategies were mainly driven by increasing template coverage. While similarity fluctuated, minimum coverage showed a clear stepwise increase, ranging from 0% to 3% across stricter selection strategies. Hence, low coverages are accepted, but at least a certain level of similarity is required. However, template coverage increased sharply at FDR1 and at the maximum F1 score, from roughly 7% to 22%. These increases varied substantially across different yields, indicating that the FDR1 and maximum F1 settings are less stable.

The availability of species abundances in the ground truth allowed to establish LODs (Fig. 5). As previously noted, species with low relative abundances are less likely to be detected, but their detection increased with higher yields (Fig. 3). Therefore, without template ID filtering, all species with a relative abundance of at least 0.1% were detected, providing an LOD of 0.1% for all yields, except the lower yield 200M that had a LOD of 1%. However, low-abundance species generally also exhibited lower template ID (Supplementary Figure S6). Because stricter FDR settings and higher sequencing yields resulted in higher template ID thresholds, these low-abundance species are consequently more prone to removal. For FDR5, the LOD increased to 1% at 500M compared to no filtering, but remained 0.1% at a minimal yield of 1000M, indicating that despite higher thresholds at higher sequencing yields, they still resulted in superior LODs compared to lower yields. Both the FDR1 and maximum F1 selection strategies had their LOD increased to 1% however, as even high sequencing yields were not enough to maintain a LOD of 0.1%.

A validation step was performed using an independent dataset of newly sequenced PDMCs to assess the taxonomic classification pipeline. These PDMCs were selected because their ground truth composition is usually well described on the labels, they often contain multiple species, and their production is subjected to strict quality control to prevent contamination, as required under European Regulation (EC) 852/2004 [54]. Additionally, an independent Illumina sequencing step and independent previously published classification pipeline were employed to ensure ground truth composition of these samples [40]. During validation with the nanopore PDMCs, the novel approach demonstrated superior performance compared to the standard approach, as observed for the DMCs (Figure S3). Moreover, application of the DMC-derived thresholds also demonstrated a considerable boost in performance (Fig. 6A). The strong boost in recall could likely be explained by the fact that probiotic species are well-characterized organisms. Before a strain is marked as probiotic, it needs to undergo a comprehensive evaluation process [55]. As a result, probiotic species are well-studied organisms and tend to have a stronger representation in reference databases with high-quality sequences or complete genomes. Additionally, although the species abundances in the PDMCs were not always specified, in the cases where they were known, and considering the number of mapped reads with KMA across all samples, no species occurred at very low relative abundances, thereby reducing the likelihood of FNs.

Although the optimized taxonomic classification pipeline was validated using the PDMCs, we hypothesized that optimization tailored towards specific use cases, such as the PDMCs could improve performance. The workflow was originally optimized on a diverse set of DMCs aiming to create a broadly applicable solution. However, previous work has shown that optimizing the workflow for specific applications, such as detecting *Bacillus* species in food enzymes, can achieve very high sensitivity and specificity [7]. Therefore, the optimization process was repeated using the PDMCs as ground truth species, adjusting template ID thresholds to create a pipeline specifically optimized for this type of sample. This tailored approach indeed demonstrated superior performance (Fig. 6B). The results demonstrated that despite the high performance of the broadly applicable taxonomic classification pipeline in this study, adaptation and optimization to specific case studies and application scopes can still result in higher performance gains. In practice, such optimization mainly involves refining the template ID thresholds using representative ground truth samples from the new sample type (e.g., wastewater, food, …), while keeping the core workflow components including post-processing steps unchanged. However, this level of optimization is only feasible when suitable ground truth samples are available, which may not always be the case for every application.

Our proposed pipeline, KAPTAIN, is not the first tool to improve performance through filtering. Bracken, while not a filtering tool itself, is a companion tool to Kraken2 that re-estimates sequence abundance, after which abundance-based thresholds can be applied [41,56]. CCMetagen is a companion tool to KMA that filters results based on depth, breadth of coverage and query identity; and additionally applies a lowest common ancestor approach based on the query identity [57]. Our previous work showed that Bracken did not consistently improve performance, and that the default CCMetagen filters were too strict [18]. Using the recommended sequence abundance thresholds from our earlier study, Bracken achieved a precision of 61.11% and recall of 85%, while CCMetagen reached a precision of 94.29% and recall of 55%. Although a direct comparison is limited by differences in the underlying reference database, DMCs, and yields, KAPTAIN achieved at FDR5 a precision of 95% and recall of 91.62%. The improved performance likely stems from the post-processing steps and the use of the template ID. Unlike Bracken and CCMetagen, KAPTAIN removes plasmid hits, which previously led to higher performance. More importantly, KAPTAIN uses template ID as a filtering metric. Both this study and a previous study [7] demonstrated template ID to be a better filter metric than sequence abundance. Applying a template ID threshold for Bracken would not be straightforward, or may even be infeasible, because it uses a spaced k-mer approach and stores only LCA information of each k-mer. For CCMetagen, since the default settings were already strict, additional filtering would only further decrease recall. However, CCMetagen’s performance could potentially be improved by refining the query identity metric or basing the LCA approach on template ID. Likewise, for KAPTAIN, future work could explore combining template ID with additional metrics to further improve performance.

We acknowledge the following limitations of our study. Firstly, for both the DMCs and the PDMCs, the matrix was still relatively clean, such that no host fraction or other sources of contaminations interfered with identification of the present species [58]. In contrast, more complex sample types such as environmental samples or host associated matrices (gut or saliva microbiomes) represent additional analytical challenges. For example, soil is a notoriously challenging sample type, as humic acids and metal ions can damage nanopore proteins and ultimately compromise sequencing quality [59], while for gut or saliva microbiomes the large human host fraction can decrease sensitivity for microbial read detection [60]. Furthermore, both soil and host microbiomes are characterized by immense microbial diversity and host DNA complexity, which can further complicate accurate taxonomic resolution [61]. The samples used in this study largely contained well-studied species. For samples with high microbial diversity, more species will be absent from the database. The obtained performance of our pipeline may therefore not be applicable to such challenging sample types. However, some of these problems could partially be mitigated by for example depleting human DNA with nanopore adaptive sampling [62] or using specialized soil kits [63]. Secondly, the number of DMCs used to optimize the workflow remains limited. Therefore, with regard to general usability, the workflow could benefit from more extensive optimization and validation. To our knowledge, this study includes more DMCs than previous studies, and no additional DMCs appear to be currently available. Furthermore, other studies rarely incorporate independent validation datasets, whereas our study includes ten PDMCs sequenced as validation datasets. However, if more DMCs become available in the future, the optimization can be further refined and more robustly validated using a larger set of DMCs. In this context, KAPTAIN can be used in a user-friendly manner to refine optimization. Thirdly, potential technical contaminations introduced during sample collection, library preparation and sequencing, such as those originating from extraction kits and reagents (“kitome”), were not taken into account as our analysis relied on publicly available data for the optimization. Such contaminations are commonly encountered in metagenomics analysis [23], and some detected species classified as FP may in fact have been really present in the samples potentially leading to an underestimation of the precision. Fourthly, our study primarily focused on the use of KMA as underlying taxonomic classifier although numerous alternative classifiers exist that may potentially even exhibit higher performance when applying the optimization strategy of our study. Although template ID is a metric specific to KMA, it represents a composite metric of both template coverage and sequence ID, which are reported by some other classifiers as well and can therefore also be implemented. Fifthly, the pipeline is specific to nanopore sequencing data and is therefore likely not directly applicable to other sequencing platforms. In particular, it was not optimized to the more widely used Illumina sequencing technology. Sixthly, our pipeline filters out viral sequences by default, and can therefore not be applied to the detection of viral species in metagenomics datasets. A similar optimization step could, however, be envisaged for virus identification, but would require the construction of DMCs containing viral species, which remains technically challenging.

## Conclusion

We present an optimized taxonomic classification pipeline for nanopore sequencing data that is broadly applicable and demonstrates superior performance compared to employing different taxonomic classifiers in default modes combined with abundance filtering, which is the current state-of-the-art. While the pipeline is broadly applicable across a range of relatively clean matrices and moderate complexity, its performance in more complex or host-associated samples (e.g., soil, gut, or saliva microbiomes) may require a new optimization and additional validation. The pipeline can be executed via the command line (https://github.com/BioinformaticsPlatformWIV-ISP/KAPTAIN) or on the Galaxy instance of our institute (https://galaxy.sciensano.be) [43]. After defining a selection strategy, and optionally specifying a target yield for downsampling, the tool automatically determines the optimal thresholds following taxonomic classification. We further anticipate that additional performance boosts may be especially reliant on optimizing the reference databases used for taxonomic classification, as this was identified as the predominant source of error.

## Supplementary Information

Supplementary Material 1.

## Data Availability

The Illumina and Nanopore sequencing data of the ten PDMCs have been deposited in the Sequence Read Archive under BioProject accession number PRJNA1334416. The database used in this study is available on Zenodo (10.5281/zenodo.17435876). The optimized taxonomic classification workflow is accessible on GitHub (https://github.com/BioinformaticsPlatformWIV-ISP/KAPTAIN) and is additionally available for execution via our Galaxy instance (https://galaxy.sciensano.be).

## Footnotes

1
https://bitbucket.org/genomicepidemiology/kma/issues/36/indexing-computational-requirements?iframe=true&spa=0#comment-60585015.

## Abbreviations

ANI: Average nucleotide identity
bp: Base pairs
DMC: Defined mock community
FDR: False discovery rate
FN: False negative
FP: False positive
KAPTAIN: KMA-bAsed Pipeline for meTAgenomic specIes ideNtification
LOD: Limit of detection
ONT: Oxford Nanopore Technologies
PDMC: Probiotic-derived mock community
Template ID: Template identity
TP: True positive
