# Supplementary material for: Filtering for truth: high-precision taxonomic classification in nanopore shotgun metagenomics data through a KMA-based bioinformatic pipeline (KAPTAIN)
Source: BMC Genomics. 2026 Feb 24;27:326. doi: 10.1186/s12864-026-12668-0 (PMC13037218; doi:10.1186/s12864-026-12668-0)
Supplement: Supplementary file 1 — Supplementary Material 1. [file 12864_2026_12668_MOESM1_ESM.docx]

# Text

## DMC composition

The same nine DMCs with public nanopore sequencing data available from an earlier study were used ^1^, with minor modifications and the addition of one DMC. Specifically, two DMCs with ONT R9 chemistry were replaced by ONT R10 chemistry and one DMC with R10 chemistry was added. The samples included a mix of: solely bacteria; bacteria with fungi; bacteria with archaea; or all three groups together. Their complexity ranged from simple with only 10 different species to very complex with over 60 species. Species were distributed in three ways: even (all species equally abundant), staggered (different abundances without a constant factor, although equal abundances occurred) or logarithmic (different abundances with each species one-tenth as abundant as the previous one). Hence, the DMCs provide a broad representation of possible metagenomic samples, excluding viruses.

## PDMC composition

The products were purchased either online or from local stores and sequenced in-house. According to their labels, the products contained between one and fourteen species (Supplementary Table S8). The labeled species composition was used as the reference truth.

## DMCs and database taxonomic consistency

To ensure taxonomic consistency between the DMCs and the employed reference database, all species names were updated with TaxonKit 0.18.0 ^2^ to reflect the taxonomy as of February 2023, when the reference database was created. For nearly all organisms in the DMCs, genome assemblies were available on NCBI, where an automatic taxonomy check based on the average nucleotide ID is performed ^3^. In some cases, the submitted name of the assembly did not match the taxonomy of NCBI’s check. In such cases, species names in our datasets were corrected to match the taxonomy of NCBI’s check. A full overview of updated species names can be found in Supplementary Table S2, with further detail in the Supplementary Text.

## PDMCs and database taxonomic consistency

Species names were harmonized according to the NCBI taxonomy (as of February 2023; see Section 1.3). Updated species names are provided in Supplementary Table S8. As no genome assemblies were available for these strains, potential taxonomic misannotations could not be corrected through NCBI’s taxonomy check, though some names were manually updated (see Supplementary Text).

## Abundance conversion

The abundance of the three strain madness datasets was in the original study described as taxonomic abundance (see Table S2). To convert this to sequence abundance, the taxonomic abundance of each genome was multiplied with its genome length. The relative sequence abundance was then calculated by dividing each sequence abundance by the total sequence abundance. The abundance of sample HM 276D was expressed in rRNA operon counts; 1,000,000 copies per genome. The rRNA operon abundance was first converted to taxonomic abundance by dividing each species’ original abundance by its corresponding average RNA operon copy number, gathered from the *rrn*DB ^4^. Afterwards, the sequence abundance of each genome was calculated by multiplying the taxonomic abundance with its genome length. The relative sequence abundance was then calculated by dividing each sequence abundance by the total sequence abundance. Sample HM 277D contained 10,000 to 10,000,000 copies per organism. However, because the specific copy numbers per species were not disclosed, this sample was excluded from the LOD analysis. Other samples were originally expressed in sequence abundance, so no conversion was necessary.

## Update ground truth

Due to the changing nature of nomenclature, previously assigned names may no longer be accurate. Since the database was built at a different time than the ground truth labels were defined, taxonomic updates can lead to inaccurate or misleading assignments. These updates generally fall into two types.

The first, and simplest, is a straightforward name change, which can be resolved by updating the ground truth label. For example, the ground truth of Zymo D6300 states *Lactobacillus fermentum,* but was changed to *Limosilactobacillus fermentum* in 2020. As such, the labels of each DMC and probiotic-derived mock community were updated to reflect the taxonomy as of February 2023.

The second is more complex and involves the splitting of an existing species into two or more distinct species. For example, *Bacillus spizizenii* is a fairly recent species from 2020, which used to be named *Bacillus subtilis*. In such cases, the associated genomes must be re-evaluated to determine whether they belong to the original species or should be reassigned to the newly defined one. For re-evaluation, the automatic taxonomy check of NCBI was used ^3^. Both types of updates are denoted in separate columns of Table S3. An exception was the *Pseudomonas fluorescens* genome (GCA_000009225, full name *Pseudomonas [fluorescens] SBW25*) in the strain madness datasets. A BLAST search revealed an almost identical match to a *Pseudomonas marginalis* genome (GCF_034424355, full name *Pseudomonas marginalis SBW25*). This *P. marginalis* passed NCBI’s taxonomy check. As a result, *P. fluorescens* was updated to *P. marginalis.*

For the probiotic-derived mock communities, re-evaluation of the species was more difficult as species in the probiotic-derived mock communities are often proprietary and their genomes are not readily available. Although an automated check through NCBI’s taxonomy check was therefore not possible, a few species names were still manually updated. The strain *Lactobacillus casei* PXN^®^ 37™ from sample PROBIOTIC 8 is not listed on NCBI. However, a literature search indicates that this strain is also known as *Lactobacillus casei* NCIMB 30185 ^5,6^, which updated to the taxonomy of February 2023 should be *Lacticaseibacillus* *casei* NCIMB 30185. Given that another study proposed reclassifying it as *Lacticaseibacillus* paracasei ^7^ and our results strongly support this classification, the name was updated accordingly to *Lacticaseibacillus* paracasei. The sample PROBIOTIC 10 contained *Lacticaseibacillus casei* R0215. Although the strain is not described in literature, other probiotic products describe the strain as *Lacticaseibacillus paracasei* R0215, even other probiotic products from same brand as PROBIOTIC 10. Additionally, a BLAST search of the sample to both species shows the majority of the reads mapping to *L. paracasei* (results not shown). Based on this information, along with our own classification results, *Lacticaseibacillus casei* in PROBIOTIC 10 was reclassified as *Lacticaseibacillus paracasei*.

## Validation of the standard versus the novel approach using PDMCs

Figure S3 compares the standard and novel approaches by showing the median precision, recall, and F1 scores across ten PDMCs under three selection strategies (no thresholds applied, maximum F1 score, and FDR5) and five sample yields. Without any filtering applied, precision declined as yield increased, with values of 83.77% and 55.77% for the novel approach, and 45% and 22.5% for the standard approach, at 200M and 2000M, respectively. The median precision of the standard approach was substantially lower than that of the novel approach, largely due to the detection of many plasmids, which, unlike chromosomal DNA, are typically not species-specific. In contrast, for both approaches, the recall of all PDMCs was 100% across all yields, indicating that all species present in the ground truth were detected. Consequently, the median F1 scores decreased as yields increased, with lower scores for the standard compared to the novel approach, with values of 61.90% and 91.15% at 200M, and 36.67% and 71.43% at 2000M, respectively.

Using a maximum F1 score selection strategy, the median precision increased for both approaches. Both approaches achieved perfect median precision, recall, and consequently also F1 scores, of 100% across all yields. However, when not considering solely median values, one sample (PROBIOTIC8) did not reach this perfect score of 100% recall when using the standard approach, which was not the case for the novel approach.

Lastly, using FDR5 as selection strategy, the observed performance was similarly higher for the PDMCs compared to the DMCs. The standard and novel approaches reached a median precision of 96.15% and 100% across all yields, respectively, although two samples displayed notably lower precision values. For both approaches, a median recall of 100% across all yields was reached. This resulted in corresponding median F1 scores of 98% for the standard approach and 100% for the novel approach across all yields.

In conclusion, as with the DMCs, the novel approach performed better for all three selection strategies compared to the standard approach.

# Tables

## Table S1

Table S1: Sources and information on the ten DMC. The first, second, and third columns respectively present the DMC name, the company or authors responsible for producing the DMC, and its catalog number (if applicable). The fourth, fifth, and seventh columns indicate the Nanopore instrument used, the pore chemistry version (along with the kit used, if available), and the software used for basecalling, respectively. The eighth and ninth columns present the source of the DMCs and their corresponding ENA accession numbers, respectively.

| Name | Source | Catalog | Instrument | Chemistry version | Basecaller version | Composition | Link | Run accession |
| --- | --- | --- | --- | --- | --- | --- | --- | --- |
| Zymo D6300 | Zymo Research | D6300 | GridION | R9 | Guppy v2.2.2 | 8 bacteria; 2 fungi | <https://doi.org/10.1093/gigascience/giz043> | ERR2906227 |
| Zymo D6310 | Zymo Research | D6310 | GridION | R9 | Guppy v2.2.2 | 8 bacteria; 2 fungi | <https://doi.org/10.1093/gigascience/giz043> | ERR2906229 |
| Zymo D6322 | Zymo Research | D6322 | PromethION | R10.4.1 LSK114 | Dorado 0.7.3 | 7 bacteria 1 fungus | <https://github.com/Kirk3gaard/MicroBench> | ERR14251398 |
| Zymo D6331 | Zymo Research | D6331 | PromethION | R10.4.1 SQK-NBD114.24 | Dorado 0.8.2 | 1 archaea; 14 bacteria; 2 fungi | <https://doi.org/10.1186/s40168-022-01415-8> | SRR17913200 |
| Zymo D6332 | Zymo Research | D6332 | PromethION | R10.4.1 SQK-NBD114.24 | Dorado 0.8.2 | 12 bacteria | <https://github.com/Kirk3gaard/MicroBench> | ERR14789832 |
| Bei Resources HM-276D | Bei Resources | HM-276D | GridION | R9.4 SQK-LSK109 | NA | 20 bacteria | <https://doi.org/10.1016/j.isci.2020.101223> | SRR11700265 |
| Bei Resources HM-277D | Bei Resources | HM-277D | GridION | R9.4 SQK-LSK109 | NA | 20 bacteria | <https://doi.org/10.1016/j.isci.2020.101223> | SRR11700264 |
| Strain madness 1 | Meslier, V., Quinquis, B., Da Silva, K. et al. | / | NA | R9 SQK-LSK109 | Guppy 2.3.1 | 22 archaea; 45 bacteria | <https://doi.org/10.1038/s41597-022-01762-z> | ERR9765780 |
| Strain madness 2 |  |  |  |  |  | 22 archaea; 61 bacteria |  | ERR9765781 |
| Strain madness 3 |  |  |  |  |  | 14 archaea; 46 bacteria |  | ERR9765782 |

## Table S2

Table S2: Species information of the ten DMCs. The first column denotes the DMC name. The second column shows the NCBI’s assembly accession of the species in the DMCs. The third column represents the original species name, while the fourth and fifth columns represent the updated species name as explained under 1.6 Update ground truth (NA means the species name was not updated). The last column shows the relative abundance (%) of the present species in the DMC(s).

|  | **NCBI Reference Genome Accession No.** | **Original species name** | **Species name on 02/2023** | **Changed species name** | **Genomic DNA (%)** | | | |
| --- | --- | --- | --- | --- | --- | --- | --- | --- |
| Zymo D6300 | GCF_028743795 | *Bacillus subtilis* | NA | *Bacillus spizizenii* | 12 | | | |
|  | GCA_028975465 | *Cryptococcus neoformans* | NA | NA | 2 | | | |
|  | GCF_028743535 | *Enterococcus faecalis* | NA | NA | 12 | | | |
|  | GCF_028743555 | *Escherichia coli* | NA | NA | 12 | | | |
|  | GCF_030770375 | *Lactobacillus fermentum* | *Limosilactobacillus fermentum* | NA | 12 | | | |
|  | GCF_028743575 | *Listeria monocytogenes* | NA | NA | 12 | | | |
|  | GCF_028743595 | *Pseudomonas aeruginosa* | NA | NA | 12 | | | |
|  | GCA_028975445 | *Saccharomyces cerevisiae* | NA | NA | 2 | | | |
|  | GCF_028743635 | *Salmonella enterica* | NA | NA | 12 | | | |
|  | GCF_028743615 | *Staphylococcus aureus* | NA | NA | 12 | | | |
| Zymo D6310 | GCF_028743795 | *Bacillus subtilis* | NA | *Bacillus spizizenii* | 0.89 | | | |
|  | GCA_028975465 | *Cryptococcus neoformans* | NA | NA | 0.00089 | | | |
|  | GCF_028743535 | *Enterococcus faecalis* | NA | NA | 0.00089 | | | |
|  | GCF_028743555 | *Escherichia coli* | NA | NA | 0.089 | | | |
|  | GCF_030770375 | *Lactobacillus fermentum* | *Limosilactobacillus fermentum* | NA | 0.0089 | | | |
|  | GCF_028743575 | *Listeria monocytogenes* | NA | NA | 89.1 | | | |
|  | GCF_028743595 | *Pseudomonas aeruginosa* | NA | NA | 8.9 | | | |
|  | GCA_028975445 | *Saccharomyces cerevisiae* | NA | NA | 0.89 | | | |
|  | GCF_028743635 | *Salmonella enterica* | NA | NA | 0.089 | | | |
|  | GCF_028743615 | *Staphylococcus aureus* | NA | NA | 0.000089 | | | |
| Zymo D6322 | GCF_028743795 | *Bacillus subtilis* | NA | *Bacillus spizizenii* | 14 | | | |
|  | GCF_028743535 | *Enterococcus faecalis* | NA | NA | 14 | | | |
|  | GCF_028743555 | *Escherichia coli* | NA | NA | 14 | | | |
|  | GCF_028743575 | *Listeria monocytogenes* | NA | NA | 14 | | | |
|  | GCF_028743595 | *Pseudomonas aeruginosa* | NA | NA | 14 | | | |
|  | GCA_028975445 | *Saccharomyces cerevisiae* | NA | NA | 2 | | | |
|  | GCF_028743635 | *Salmonella enterica* | NA | NA | 14 | | | |
|  | GCF_028743615 | *Staphylococcus aureus* | NA | NA | 14 | | | |
| Zymo D6331 | GCF_028743255 | *Akkermansia muciniphila* | NA | NA | 1.5 | | | |
|  | GCF_028743275 | *Bacteroides fragilis* | NA | NA | 14 | | | |
|  | GCF_028743295 | *Bifidobacterium adolescentis* | NA | NA | 6 | | | |
|  | NA | *Candida albicans* | NA | NA | 1.5 | | | |
|  | GCF_028743315 | *Clostridioides difficile* | NA | NA | 1.5 | | | |
|  | GCF_028743735 | *Clostridium perfringens* | NA | NA | 0.0001 | | | |
|  | NA | *Enterococcus faecalis* | NA | NA | 0.001 | | | |
|  | GCF_028743355 GCF_028743335 GCF_028743755 GCF_028743555 GCF_028743375 | *Escherichia coli* | NA | NA | 14 | | | |
|  | GCF_028743395 | *Faecalibacterium prausnitzii* | NA | NA | 14 | | | |
|  | GCF_028743415 | *Fusobacterium nucleatum* | NA | NA | 6 | | | |
|  | GCF_028743095 | *Lactobacillus fermentum* | *Limosilactobacillus fermentum* | NA | 6 | | | |
|  | GCF_028743435 | *Methanobrevibacter smithii* | NA | NA | 0.1 | | | |
|  | GCF_028743775 | *Prevotella corporis* | NA | NA | 6 | | | |
|  | GCF_028743455 | *Roseburia hominis* | NA | NA | 14 | | | |
|  | GCA_030867715 | *Saccharomyces cerevisiae* | NA | NA | 1.4 | | | |
|  | GCF_028743635 | *Salmonella enterica* | NA | NA | 0.01 | | | |
|  | GCF_028743475 | *Veillonella rogosae* | NA | NA | 14 | | | |
| Zymo D6332 | GCF_031191795 | *Fusobacterium nucleatum* | NA | NA | 1 | | | |
|  | GCF_031191205 | *Haemophilus parainfluenzae* | NA | NA | 8 | | | |
|  | GCF_031190635 | *Neisseria subflava* | NA | NA | 16 | | | |
|  | GCF_031323855 | *Porphyromonas gingivalis* | NA | NA | 1 | | | |
|  | GCF_031191185 | *Prevotella nigrescens* | NA | NA | 16 | | | |
|  | GCF_031191775 | *Rothia dentocariosa* | NA | NA | 8 | | | |
|  | GCF_031191545 | *Schaalia odontolytica* | NA | NA | 8 | | | |
|  | GCF_031191225 | *Streptococcus mitis* | NA | NA | 8 | | | |
|  | GCF_044360185 | *Streptococcus mutans* | NA | NA | 1 | | | |
|  | GCF_031582885 | *Streptococcus parasanguinis* | NA | NA | 16 | | | |
|  | GCF_031192325 | *Streptococcus salivarius* | NA | NA | 1 | | NA | |
|  | GCF_031190775 | *Veillonella parvula* | NA | NA | 16 | | NA | |
| HM 276D HM 277D | GCF_000015425 | *Acinetobacter baumannii* | NA | NA | 4.86 | | NA | |
|  | GCF_000154225 | *Actinomyces odontolyticus* | *Schaalia odontolytica* | *Schaalia dentiphila* | 5.78 | | NA | |
|  | GCF_000008005 | *Bacillus cereus* | NA | *Bacillus pacificus* | 2.87 | | NA | |
|  | GCF_000012825 | *Bacteroides vulgatus* | *Phocaeicola vulgatus* | NA | 5.34 | | NA | |
|  | GCF_000016965 | *Clostridium beijerinckii* | NA | NA | 2.86 | | NA | |
|  | GCF_000008565 | *Deinococcus radiodurans* | NA | NA | 7.93 | | NA | |
|  | GCF_000172575 | *Enterococcus faecalis* | NA | NA | 4.99 | | NA | |
|  | GCF_000005845 | *Escherichia coli* | NA | NA | 4.82 | | NA | |
|  | GCF_000008525 | *Helicobacter pylori* | NA | NA | 6.14 | | NA | |
|  | GCF_000014425 | *Lactobacillus gasseri* | NA | NA | 2.7 | | NA | |
|  | GCF_000196035 | *Listeria monocytogenes* | NA | NA | 3.77 | | NA | |
|  | GCF_000008805 | *Neisseria meningitidis* | NA | NA | 4.1 | | NA | |
|  | GCF_000008345 | *Propionibacterium acnes* | *Cutibacterium acnes* | NA | 6.08 | | NA | |
|  | GCF_000006765 | *Pseudomonas aeruginosa* | NA | NA | 11.36 | | NA | |
|  | GCF_000012905 | *Rhodobacter sphaeroides* | *Cereibacter sphaeroides* | NA | 10.26 | | NA | |
|  | GCF_000017085 | *Staphylococcus aureus* | NA | NA | 3.66 | | NA | |
|  | GCF_000007645 | *Staphylococcus epidermidis* | NA | NA | 3.3 | | NA | |
|  | GCF_000007265 | *Streptococcus agalactiae* | NA | NA | 2.25 | | NA | |
|  | GCF_000007465 | *Streptococcus mutans* | NA | NA | 2.94 | | NA | |
|  | GCF_000006885 | *Streptococcus pneumoniae* | NA | NA | 4.0 | | NA | |
| Strain Madness 1 Strain Madness 2 Strain Madness 3 | GCA_000144915 | *Acidilobus saccharovorans* | NA | NA | 0.0183 | 0.018 | | 0.0182 |
|  | GCA_000022565 | *Acidobacterium capsulatum* | NA | NA | 0.2967 | 0.2924 | | 0.1825 |
|  | GCA_000025665 | *Aciduliprofundum boonei* | *Candidatus Aciduliprofundum boonei* | NA | 0.0183 | 0.018 | | 0.0 |
|  | GCA_000015425 | *Acinetobacter baumannii* | NA | NA | 0.0 | 0.0722 | | 0.0 |
|  | GCA_000020225 | *Akkermansia muciniphila* | NA | NA | 0.8699 | 0.8573 | | 1.2136 |
|  | GCA_000008665 | *Archaeoglobus fulgidus* | NA | NA | 0.9157 | 0.9025 | | 0.73 |
|  | GCA_000008005 | *Bacillus cereus* | NA | NA | 0.0 | 0.0722 | | 0.0 |
|  | GCA_000011065 | *Bacteroides thetaiotaomicron* | NA | NA | 0.7325 | 0.722 | | 0.4562 |
|  | GCA_000010425 | *Bifidobacterium adolescentis* | NA | NA | 0.0 | 0.0722 | | 0.0 |
|  | GCA_000195675 | *Bordetella pertussis* | *Bordetella bronchiseptica* | NA | 0.9157 | 0.9025 | | 0.9125 |
|  | GCA_000022325 | *Caldicellulosiruptor bescii* | NA | NA | 0.2253 | 0.222 | | 0.2245 |
|  | GCA_000016545 | *Caldicellulosiruptor saccharolyticus* | NA | NA | 0.9523 | 0.9386 | | 1.4235 |
|  | GCA_000015985 | *Cereibacter A* | *Cereibacter sphaeroides* | NA | 0.0 | 0.0722 | | 0.0 |
|  | GCA_000006985 | *Chlorobaculum tepidum* | NA | NA | 0.1886 | 0.1859 | | 0.4708 |
|  | GCA_000020465 | *Chlorobium limicola* | NA | NA | 2.3075 | 2.2742 | | 1.3797 |
|  | GCA_000015125 | *Chlorobium phaeobacteroides* | NA | NA | 0.7783 | 0.7671 | | 0.7756 |
|  | GCA_000016085 | *Chlorobium phaeovibrioides* | NA | NA | 0.9157 | 0.9025 | | 1.3687 |
|  | GCA_000018865 | *Chloroflexus aurantiacus* | NA | NA | 0.3846 | 0.379 | | 0.1916 |
|  | GCA_000767745 | *Clostridium beijerinckii* | NA | NA | 0.0 | 0.0722 | | 0.0 |
|  | GCA_000231215 | *Cutibacterium acnes* | NA | NA | 0.0 | 0.0722 | | 0.0 |
|  | GCA_000008565 | *Deinococcus radiodurans* | NA | NA | 0.7325 | 0.7931 | | 0.5475 |
|  | GCA_000243155 | *Desulfitobacterium dehalogenans* | NA | NA | 0.0055 | 0.0054 | | 0.0 |
|  | GCA_002952055 | *Desulfobulbus oralis* | NA | NA | 7.3253 | 7.2196 | | 3.65 |
|  | GCA_000023225 | *Desulfomicrobium baculatum* | NA | NA | 0.0 | 0.0 | | 0.0547 |
|  | GCA_000189295 | *Desulfovibrio mercurii* | NA | NA | 0.0733 | 0.0722 | | 0.0456 |
|  | GCA_000156375 | *Desulfovibrio piger* | NA | NA | 4.5783 | 4.5123 | | 6.8437 |
|  | GCA_000021645 | *Dictyoglomus turgidum* | NA | NA | 0.4578 | 0.4512 | | 0.365 |
|  | GCA_000172575 | *Enterococcus faecalis* | NA | NA | 0.1831 | 0.2516 | | 0.365 |
|  | GCA_000005845 | *Escherichia coli* | NA | NA | 0.0 | 0.0722 | | 0.0 |
|  | GCA_003019295 | *Fusobacterium nucleatum* | NA | NA | 2.1976 | 2.1659 | | 1.095 |
|  | GCA_000010305 | *Gemmatimonas aurantiaca* | NA | NA | 4.0289 | 3.9708 | | 4.015 |
|  | GCA_000020725 | *Geobacter bemidjiensis* | NA | NA | 0.0055 | 0.0054 | | 0.0 |
|  | GCA_000007985 | *Geobacter sulfurreducens* | NA | NA | 0.8058 | 0.7942 | | 0.1405 |
|  | GCA_000006805 | *Halobacterium salinarum* | NA | NA | 1.8313 | 1.8049 | | 1.825 |
|  | GCA_000025685 | *Haloferax volcanii* | NA | NA | 0.0494 | 0.0487 | | 0.0493 |
|  | GCA_000008525 | *Helicobacter pylori* | NA | NA | 0.0 | 0.0722 | | 0.0 |
|  | GCA_000018565 | *Herpetosiphon aurantiacus* | NA | NA | 4.0289 | 3.9708 | | 6.4239 |
|  | GCA_000020785 | *Hydrogenobaculum sp.* | *Hydrogenobaculum sp. Y04AAS1* | NA | 0.9157 | 0.9025 | | 0.73 |
|  | GCA_000017945 | *Ignicoccus hospitalis* | NA | NA | 1.3277 | 1.3086 | | 1.9838 |
|  | GCA_001481685 | *Ignicoccus islandicus* | NA | NA | 0.5494 | 0.5415 | | 1.3687 |
|  | GCA_000014425 | *Lactobacillus gasseri* | NA | NA | 0.0 | 0.0722 | | 0.0 |
|  | GCA_000019785 | *Leptothrix cholodnii* | NA | NA | 0.0879 | 0.0866 | | 0.0 |
|  | GCA_001639275 | *Methanobrevibacter oralis* | NA | NA | 0.586 | 0.5776 | | 0.0 |
|  | GCA_000091665 | *Methanocaldococcus jannaschii* | NA | NA | 3.5345 | 3.4835 | | 7.0444 |
|  | GCA_000016125 | *Methanococcus maripaludis* | NA | NA | 0.7508 | 0.74 | | 0.7482 |
|  | GCA_000011585 | *Methanococcus maripaludis* | NA | NA | 0.2747 | 0.2707 | | 0.1825 |
|  | GCA_000308215 | *Methanomassiliicoccus luminyensis* | NA | NA | 0.0549 | 0.0541 | | 0.0 |
|  | GCA_000328665 | *Methanomethylovorans hollandica* | NA | NA | 0.0458 | 0.0451 | | 0.0 |
|  | GCA_000007185 | *Methanopyrus kandleri* | NA | NA | 0.1282 | 0.1263 | | 0.1606 |
|  | GCA_000007345 | *Methanosarcina acetivorans* | NA | NA | 0.3663 | 0.361 | | 0.365 |
|  | GCA_000166095 | *Methanothermus fervidus* | NA | NA | 3.2048 | 3.1586 | | 2.555 |
|  | GCA_000018265 | *Micromonospora arenicola* | *Salinispora arenicola* | NA | 0.8131 | 0.8014 | | 0.657 |
|  | GCA_000016425 | *Micromonospora tropica* | *Salinispora tropica* | NA | 0.9157 | 0.9025 | | 0.73 |
|  | GCA_000008085 | *Nanoarchaeum equitans* | NA | NA | 0.1685 | 0.1661 | | 0.1113 |
|  | GCA_000008805 | *Neisseria meningitidis* | NA | NA | 0.0 | 0.0722 | | 0.0 |
|  | GCA_000195755 | *Nitratidesulfovibrio vulgaris* | NA | NA | 0.0366 | 0.0361 | | 0.073 |
|  | GCA_000009145 | *Nitrosomonas europaea* | NA | NA | 5.7504 | 5.6674 | | 2.8652 |
|  | GCA_000013645 | *Paraburkholderia xenovorans* | NA | NA | 2.0145 | 1.9854 | | 1.0037 |
|  | GCA_000154225 | *Pauljensenia odontolyticus* | *Schaalia dentiphila* | NA | 0.0 | 0.0722 | | 0.0 |
|  | GCA_000020645 | *Pelodictyon phaeoclathratiforme* | NA | NA | 0.0 | 0.0 | | 0.1131 |
|  | GCA_000021565 | *Persephonella marina* | NA | NA | 2.0035 | 1.9746 | | 1.095 |
|  | GCA_000012825 | *Phocaeicola vulgatus* | NA | NA | 0.8717 | 0.9298 | | 2.6061 |
|  | GCA_000010505 | *Porphyromonas gingivalis* | NA | NA | 1.8387 | 1.887 | | 3.6646 |
|  | GCA_002563335 | *Pseudomonas aeruginosa* | NA | NA | 0.0 | 0.0722 | | 0.0 |
|  | GCA_931907645 | *Pseudomonas fluorescens* | NA | *Pseudomonas marginalis* | 2.747 | 2.7074 | | 0.9125 |
|  | GCA_000007565 | *Pseudomonas putida* | NA | NA | 3.2964 | 3.2488 | | 2.6645 |
|  | GCA_000007225 | *Pyrobaculum aerophilum* | NA | NA | 0.1831 | 0.1805 | | 0.0912 |
|  | GCA_000016385 | *Pyrobaculum arsenaticum* | NA | NA | 0.0366 | 0.0361 | | 0.0 |
|  | GCA_000015805 | *Pyrobaculum calidifontis* | NA | NA | 1.8313 | 1.8049 | | 0.365 |
|  | GCA_000007305 | *Pyrococcus furiosus* | NA | NA | 0.2747 | 0.2707 | | 0.0 |
|  | GCA_000011105 | *Pyrococcus horikoshii* | NA | NA | 3.6627 | 3.6098 | | 4.5625 |
|  | GCA_000196115 | *Rhodopirellula baltica* | NA | NA | 3.388 | 3.3391 | | 2.0257 |
|  | GCA_000011965 | *Ruegeria pomeroyi* | NA | NA | 0.1099 | 0.1083 | | 0.0 |
|  | GCA_000015865 | *Ruminiclostridium thermocellum* | *Acetivibrio thermocellus* | NA | 0.3718 | 0.3664 | | 0.2226 |
|  | GCA_000017325 | *Shewanella baltica* | NA | NA | 2.5932 | 2.5558 | | 3.4456 |
|  | GCA_000021665 | *Shewanella baltica* | NA | NA | 4.8897 | 4.8191 | | 5.8472 |
|  | GCA_000016065 | *Shewanella loihica* | NA | NA | 0.9157 | 0.9025 | | 1.2775 |
|  | GCA_000013465 | *Staphylococcus aureus* | NA | NA | 0.0 | 0.0722 | | 0.0 |
|  | GCA_000007645 | *Staphylococcus epidermidis* | NA | NA | 0.0 | 0.0722 | | 0.0 |
|  | GCA_000007265 | *Streptococcus agalactiae* | NA | NA | 0.0 | 0.0722 | | 0.0 |
|  | GCA_000817065 | *Streptococcus mutans* | NA | NA | 0.0 | 0.0722 | | 0.0 |
|  | GCA_000020325 | *Sulfurihydrogenibium sp.* | *Sulfurihydrogenibium sp. YO3AOP1* | NA | 0.4578 | 0.4512 | | 0.365 |
|  | GCA_000011205 | *Sulfurisphaera tokodaii* | NA | NA | 0.0733 | 0.0722 | | 0.073 |
|  | GCA_000019085 | *Thermoanaerobacter pseudethanolicus* | NA | *Thermoanaerobacter brockii* | 2.1097 | 2.0793 | | 1.5768 |
|  | GCA_000019625 | *Thermotoga maritima* | *Thermotoga sp. RQ2* | NA | 0.0 | 0.0 | | 4.1975 |
|  | GCA_000018945 | *Thermotoga neapolitana* | NA | NA | 1.0896 | 1.0739 | | 2.1717 |
|  | GCA_000016785 | *Thermotoga petrophila* | NA | NA | 4.56 | 4.4942 | | 0.0 |
|  | GCA_000008185 | *Treponema denticola* | NA | NA | 1.2966 | 1.2779 | | 1.6151 |
|  | GCA_000009705 | *Trichormus sp.* | *Nostoc sp. PCC 7120 = FACHB-418* | NA | 3.5528 | 3.5015 | | 5.3107 |
|  | GCA_000196135 | *Wolinella succinogenes* | NA | NA | 0.4981 | 0.4909 | | 0.4143 |
|  | GCA_000007105 | *Zymomonas mobilis* | NA | NA | 0.0 | 0.0 | | 0.0365 |

## Table S3

Table S3: Sequence statistics of the DMCs before and after filtering. The first column denotes before (raw) and after (filtered) filtering. The second column shows the DMC name. The third, the fourth, the fifth, the sixth and seventh columns denote the number of sequences, the number of bases, their average Phred score, average length and N50, respectively.

|  | **DMC** | **#Sequences** | **Yield** | **AvgQual** | **AvgLen** | **N50** |
| --- | --- | --- | --- | --- | --- | --- |
| Raw | Zymo D6300 | 3,491,078 | 14,007,156,825 | 8.33 | 4,012.3 | 5,213 |
|  | Zymo D6310 | 3,667,007 | 16,032,264,247 | 7.96 | 4,372 | 5,290 |
|  | Zymo D6322 | 8,851,918 | 31,995,546,765 | 13.56 | 3,614.5 | 13,837 |
|  | Zymo D6331 | 5,757,345 | 28,443,158,238 | 13.65 | 4,940.3 | 5,913 |
|  | Zymo D6332 | 1,140,052 | 7,530,532,667 | 20.57 | 6,605.4 | 8,598 |
|  | Bei Resources HM-276D | 11,610,183 | 35,578,375,166 | 8.68 | 3,064.4 | 6,828 |
|  | Bei Resources HM-277D | 18,254,839 | 72,312,638,112 | 10.06 | 3,961.3 | 7,857 |
|  | Strain madness 1 | 696,944 | 3,125,920,499 | 10.62 | 4,485.2 | 6,086 |
|  | Strain madness 2 | 831,802 | 3,690,876,744 | 10.63 | 4,437.2 | 6,057 |
|  | Strain madness 3 | 791,715 | 3,412,736,796 | 10.6 | 4,310.6 | 5,788 |
| Filtered | Zymo D6300 | 688,666 | 3,184,351,336 | 10.38 | 4,623.9 | 5,397 |
|  | Zymo D6310 | 407,843 | 1,934,530,540 | 10.31 | 4,743.3 | 5,442 |
|  | Zymo D6322 | 4,401,162 | 28,272,769,892 | 13.95 | 6,423.9 | 15,966 |
|  | Zymo D6331 | 5,757,345 | 28,443,158,238 | 13.65 | 4,940.3 | 5,913 |
|  | Zymo D6332 | 1,076,408 | 7,468,390,687 | 20.84 | 6,938.3 | 8,627 |
|  | Bei Resources HM-276D | 2,163,256 | 11,018,419,575 | 10.52 | 5,093.4 | 8,572 |
|  | Bei Resources HM-277D | 7,002,956 | 37,506,985,368 | 11.71 | 5,355.9 | 8,560 |
|  | Strain madness 1 | 485,802 | 2,428,082,669 | 11.2 | 4,998.1 | 6,182 |
|  | Strain madness 2 | 579,446 | 2,875,480,429 | 11.2 | 4,962.5 | 6,157 |
|  | Strain madness 3 | 553,288 | 2,647,336,371 | 11.17 | 4,784.7 | 5,882 |

## Table S4

Table S4: Sequence statistics of the probiotic-derived mock communities before and after filtering. The first column denotes before (raw) and after (filtered) filtering. The second column shows the probiotic-derived mock community name. The third, the fourth, the fifth, the sixth and seventh columns denote the number of sequences, the number of bases, their average Phred score, average length and N50, respectively.

|  | **Probiotic** | **#Sequences** | **Yield** | **AvgQual** | **AvgLen** | **N50** |
| --- | --- | --- | --- | --- | --- | --- |
| Raw | PROBIOTIC1 | 3,541,293 | 19,215,502,743 | 11.72 | 5,426.1 | 12,928 |
|  | PROBIOTIC2 | 5,814,033 | 12,001,290,346 | 12.28 | 2,064.2 | 3,454 |
|  | PROBIOTIC3 | 1,375,061 | 2,356,782,880 | 12.97 | 1,713.9 | 2,463 |
|  | PROBIOTIC4 | 4,527,675 | 16,019,595,242 | 12.9 | 3,538.2 | 4,519 |
|  | PROBIOTIC5 | 751,547 | 2,490,777,975 | 12.71 | 3,314.2 | 4,423 |
|  | PROBIOTIC6 | 1,089,396 | 4,019,809,690 | 13.7 | 3,689.9 | 6,423 |
|  | PROBIOTIC7 | 1,063,709 | 2,628,339,571 | 12.04 | 2,470.9 | 3,982 |
|  | PROBIOTIC8 | 4,002,421 | 11,231,916,704 | 14.1 | 2,806.3 | 3,743 |
|  | PROBIOTIC9 | 4,150,274 | 9,860,786,912 | 13.47 | 2,375.9 | 3,331 |
|  | PROBIOTIC10 | 5,676,447 | 7,758,362,901 | 12.95 | 1,366.8 | 2,373 |
| Filtered | PROBIOTIC1 | 2,074,105 | 14,347,056,176 | 16.17 | 6,917.2 | 13,060 |
|  | PROBIOTIC2 | 2,718,591 | 8,847,033,162 | 16.05 | 3,254.3 | 3,872 |
|  | PROBIOTIC3 | 745,755 | 1,780,800,204 | 17.84 | 2,387.9 | 2,646 |
|  | PROBIOTIC4 | 3,275,806 | 13,054,825,270 | 17.41 | 3,985.2 | 4,560 |
|  | PROBIOTIC5 | 540,226 | 2,091,326,685 | 17.27 | 3,871.2 | 4,444 |
|  | PROBIOTIC6 | 742,646 | 3,496,515,149 | 17.56 | 4,708.2 | 6,540 |
|  | PROBIOTIC7 | 581,947 | 1,970,407,791 | 16.69 | 3,385.9 | 4,246 |
|  | PROBIOTIC8 | 2,852,193 | 9,608,104,802 | 18.14 | 3,368.7 | 3,834 |
|  | PROBIOTIC9 | 2,610,428 | 8,020,864,325 | 17.6 | 3,072.6 | 3,474 |
|  | PROBIOTIC10 | 1,961,931 | 5,215,493,143 | 17.15 | 2,658.3 | 3,119 |

## Table S5

Table S5: Template ID values and its corresponding template coverage and similarity for our optimized pipeline. The first and second column shows the sample yield and filter setting. The third, fourth and fifth column show the corresponding template ID, along with its template coverage and similarity.

| **Sample Yield** | **Filter setting** | **Template ID (%)** | **Template Coverage (%)** | **Similarity (%)** |
| --- | --- | --- | --- | --- |
| 200M | Maximum F1 | 6.60 | 7.11 | 95.84 |
|  | FDR 15% | 0.00 | 0.00 | 0.00 |
|  | FDR 10% | 0.10 | 0.16 | 95.13 |
|  | FDR 5% | 1.72 | 1.85 | 94.78 |
|  | FDR 1% | 8.11 | 8.65 | 94.71 |
| 500M | Maximum F1 | 2.67 | 2.84 | 94.81 |
|  | FDR 15% | 0.07 | 0.12 | 81.95 |
|  | FDR 10% | 0.15 | 0.60 | 93.60 |
|  | FDR 5% | 1.97 | 2.13 | 93.32 |
|  | FDR 1% | 10.88 | 13.44 | 93.46 |
| 1000M | Maximum F1 | 3.14 | 3.39 | 93.18 |
|  | FDR 15% | 1.06 | 1.15 | 95.50 |
|  | FDR 10% | 1.46 | 1.56 | 94.20 |
|  | FDR 5% | 2.39 | 2.90 | 84.48 |
|  | FDR 1% | 12.78 | 15.21 | 88.06 |
| 1500M | Maximum F1 | 11.16 | 12.99 | 95.66 |
|  | FDR 15% | 1.18 | 1.27 | 94.14 |
|  | FDR 10% | 2.05 | 2.27 | 95.48 |
|  | FDR 5% | 2.59 | 2.81 | 94.10 |
|  | FDR 1% | 13.71 | 14.96 | 93.39 |
| 2000M | Maximum F1 | 12.25 | 13.14 | 93.50 |
|  | FDR 15% | 1.26 | 1.47 | 87.46 |
|  | FDR 10% | 2.25 | 2.69 | 93.91 |
|  | FDR 5% | 2.75 | 3.35 | 86.00 |
|  | FDR 1% | 18.13 | 22.23 | 94.36 |

## Table S6

Table S6: DMC filtering threshold used for Figure 2, Figure 4 and Figure S1. The first column shows the filter approach. The second, third column and fourth lists the selection strategy, the sample yield and the used filter threshold, respectively. For the standard approach, the filter threshold is based on the relative abundance, while to the novel approach, the threshold is based on the Template ID.

| **Approach** | **Selection strategy** | **Sample yield** | **Threshold (%)** |
| --- | --- | --- | --- |
| Standard | No threshold | 200M | NA |
|  |  | 500M | NA |
|  |  | 1000M | NA |
|  |  | 1500M | NA |
|  |  | 2000M | NA |
|  | Maximum F1 | 200M | 0.017 |
|  |  | 500M | 0.009 |
|  |  | 1000M | 0.016 |
|  |  | 1500M | 0.015 |
|  |  | 2000M | 0.016 |
|  | FDR 5% | 200M | 0.305 |
|  |  | 500M | 0.387 |
|  |  | 1000M | 0.386 |
|  |  | 1500M | 0.384 |
|  |  | 2000M | 0.387 |
| Novel | No threshold | 200M | NA |
|  |  | 500M | NA |
|  |  | 1000M | NA |
|  |  | 1500M | NA |
|  |  | 2000M | NA |
|  | Maximum F1 | 200M | 6.595 |
|  |  | 500M | 2.674 |
|  |  | 1000M | 3.135 |
|  |  | 1500M | 11.163 |
|  |  | 2000M | 12.252 |
|  | FDR 5% | 200M | 1.716 |
|  |  | 500M | 1.968 |
|  |  | 1000M | 2.390 |
|  |  | 1500M | 2.592 |
|  |  | 2000M | 2.745 |

## Table S7

Table S 7: PDMC filtering threshold used for Figure S7B. The first and second column shows the sample yield and filter setting. The third column shows the corresponding template ID.

| **Sample Yield** | **Filter setting** | **Template ID (%)** |
| --- | --- | --- |
| 200M | Maximum F1 | 7.359 |
|  | FDR 15% | 0.003 |
|  | FDR 10% | 0.003 |
|  | FDR 5% | 1.023 |
|  | FDR 1% | 1.023 |
| 500M | Maximum F1 | 12.442 |
|  | FDR 15% | 0.019 |
|  | FDR 10% | 1.324 |
|  | FDR 5% | 1.383 |
|  | FDR 1% | 1.383 |
| 1000M | Maximum F1 | 17.258 |
|  | FDR 15% | 1.405 |
|  | FDR 10% | 1.57 |
|  | FDR 5% | 1.60 |
|  | FDR 1% | 1.60 |
| 1500M | Maximum F1 | 23.28 |
|  | FDR 15% | 1.50 |
|  | FDR 10% | 1.82 |
|  | FDR 5% | 2.44 |
|  | FDR 1% | 2.44 |
| 2000M | Maximum F1 | 26.02 |
|  | FDR 15% | 1.50 |
|  | FDR 10% | 1.88 |
|  | FDR 5% | 3.59 |
|  | FDR 1% | 3.59 |

## Table S8

Table S8: Species information of the ten probiotic-derived mock communities. The first column denotes the probiotic name The second column represents the original species name based on the product label, while the fourth column represents the updated species name as explained under 1.2 Update ground truth (NA means the species name was not updated).

| **Sample name** | **Species name** | **Species name on 02/2023** | **Changed species name** |
| --- | --- | --- | --- |
| PROBIOTIC1 | *Lactobacillus gasseri* | NA | NA |
|  | *Bifidobacterium bifidum* | NA | NA |
|  | *Bifidobacterium longum* | NA | NA |
| PROBIOTIC2 | *Lactobacillus rhamnosus* | *Lacticaseibacillus rhamnosus* | NA |
|  | *Lactobacillus acidophilus* | NA | NA |
|  | *Lactobacillus paracasei* | *Lacticaseibacillus paracasei* | NA |
|  | *Lactobacillus plantarum* | *Lactiplantibacillus plantarum* | NA |
|  | *Bifidobacterium longum* | NA | NA |
|  | *Bifidobacterium breve* | NA | NA |
| PROBIOTIC3 | *Lactobacillus crispatus* | NA | NA |
|  | *Lactobacillus acidophilus* | NA | NA |
|  | *Lactobacillus brevis* | *Levilactobacillus brevis* | NA |
| PROBIOTIC4 | *Lactobacillus reuteri* | *Limosilactobacillus reuteri* | NA |
|  | *Lactobacillus rhamnosus* | *Lacticaseibacillus rhamnosus* | NA |
| PROBIOTIC5 | *Akkermansia muciniphila* | NA | NA |
| PROBIOTIC6 | *Lactiplantibacillus pentosus* | NA | NA |
|  | *Lactiplantibacillus plantarum* | NA | NA |
| PROBIOTIC7 | *Streptococcus salivarius* | NA | NA |
| PROBIOTIC8 | *Bacillus subtilis* | NA | NA |
|  | *Bifidobacterium bifidum* | NA | NA |
|  | *Bifidobacterium breve* | NA | NA |
|  | *Bifidobacterium infantis* | *Bifidobacterium longum* | NA |
|  | *Bifidobacterium longum* | NA | NA |
|  | *Lactobacillus helveticus* | NA | NA |
|  | *Lactobacillus delbrueckii* | NA | NA |
|  | *Lactobacillus casei* | *Lacticaseibacillus casei* | *Lacticaseibacillus* paracasei |
|  | *Lactobacillus plantarum* | *Lactiplantibacillus plantarum* | NA |
|  | *Lactobacillus rhamnosus* | *Lacticaseibacillus rhamnosus* | NA |
|  | *Lactobacillus salivarius* | *Ligilactobacillus salivarius* | NA |
|  | *Lactococcus lactis* | NA | NA |
|  | *Streptococcus thermophilus* | NA | NA |
| PROBIOTIC9 | *Bifidobacterium breve* | NA | NA |
|  | *Bifidobacterium longum* | NA | NA |
|  | *Bifidobacterium infantis* | *Bifidobacterium longum* | NA |
|  | *Streptococcus thermophilus* | NA | NA |
|  | *Lactobacillus acidophilus* | NA | NA |
|  | *Lactobacillus rhamnosus* | *Lacticaseibacillus rhamnosus* | NA |
|  | *Lactobacillus plantarum* | *Lactiplantibacillus plantarum* | NA |
|  | *Lactobacillus paracasei* | *Lacticaseibacillus paracasei* | NA |
|  | *Lactobacillus gasseri* | NA | NA |
|  | *Lactobacillus bulgaricus* | *Lactobacillus delbrueckii* | NA |
| PROBIOTIC10 | *Lacticaseibacillus rhamnosus* | NA | NA |
|  | *Lactobacillus helveticus* | NA | NA |
|  | *Pediococcus acidilactici* | NA | NA |
|  | *Lacticaseibacillus casei* | NA | *Lacticaseibacillus paracasei* |
|  | *Bifidobacterium longum* | NA | NA |
|  | *Lactiplantibacillus plantarum* | NA | NA |
|  | *Bifidobacterium breve* | NA | NA |
|  | *Lactococcus lactis subsp. lactis* | *Lactococcus lactis* | NA |

## Table S9

Table S9: PDMC filtering threshold used for Figure S3. The first column shows the filter approach. The second, third column and fourth lists the selection strategy, the sample yield and the used filter threshold, respectively. For the standard approach, the filter threshold is based on the relative abundance, while to the novel approach, the threshold is based on the Template ID.

| **Approach** | **Selection strategy** | **Sample yield** | **Threshold (%)** |
| --- | --- | --- | --- |
| Standard | No threshold | 200M | NA |
|  |  | 500M | NA |
|  |  | 1000M | NA |
|  |  | 1500M | NA |
|  |  | 2000M | NA |
|  | Maximum F1 | 200M | 2.10 |
|  |  | 500M | 2.10 |
|  |  | 1000M | 2.04 |
|  |  | 1500M | 2.05 |
|  |  | 2000M | 2.03 |
|  | FDR 5% | 200M | 0.03 |
|  |  | 500M | 0.03 |
|  |  | 1000M | 0.04 |
|  |  | 1500M | 0.04 |
|  |  | 2000M | 0.04 |
| Novel | No threshold | 200M | NA |
|  |  | 500M | NA |
|  |  | 1000M | NA |
|  |  | 1500M | NA |
|  |  | 2000M | NA |
|  | Maximum F1 | 200M | 7.36 |
|  |  | 500M | 12.44 |
|  |  | 1000M | 17.26 |
|  |  | 1500M | 23.29 |
|  |  | 2000M | 26.02 |
|  | FDR 5% | 200M | 1.02 |
|  |  | 500M | 1.38 |
|  |  | 1000M | 1.60 |
|  |  | 1500M | 2.44 |
|  |  | 2000M | 3.59 |

# Figures

## Figure S1


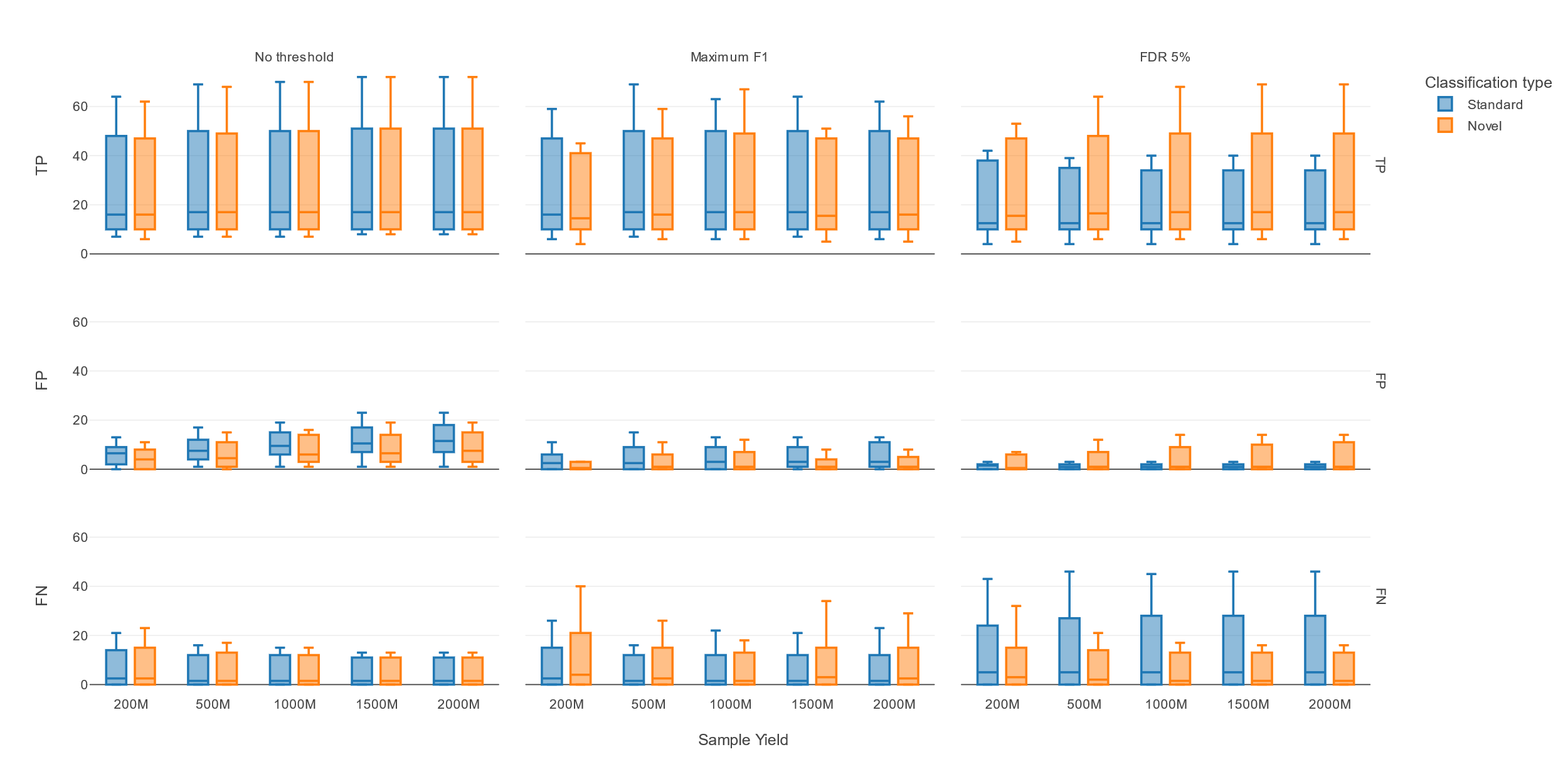


Figure S1: Number of TPs, FPs and FNs of the standard and novel approaches applied to the DMCs. Rows show the number of TPs, FPs, and FNs, while columns represent the three selection strategies: no thresholds applied, maximum F1 score, and FDR5. The x-axis shows the five sample yields, whereas the y-axis shows the respective metric values expressed as boxplots. Blue and orange boxplots indicate the performance for the standard and novel approaches, respectively, with solid and dotted lines indicating the median and mean of each boxplot. The used filter thresholds are shown in Table S6.

## Figure S2

Figure S2: MetaCARP analysis of the ten probiotic-derived mock communities. Rows show the species names and columns show the probiotic-derived mock community names. A cell filled with dark blue means a high confidence detection with MetaCARP while a light blue means a low confidence detection. Hatched cells are expected species based on the product label (i.e., species in the ground truth).

## Figure S3


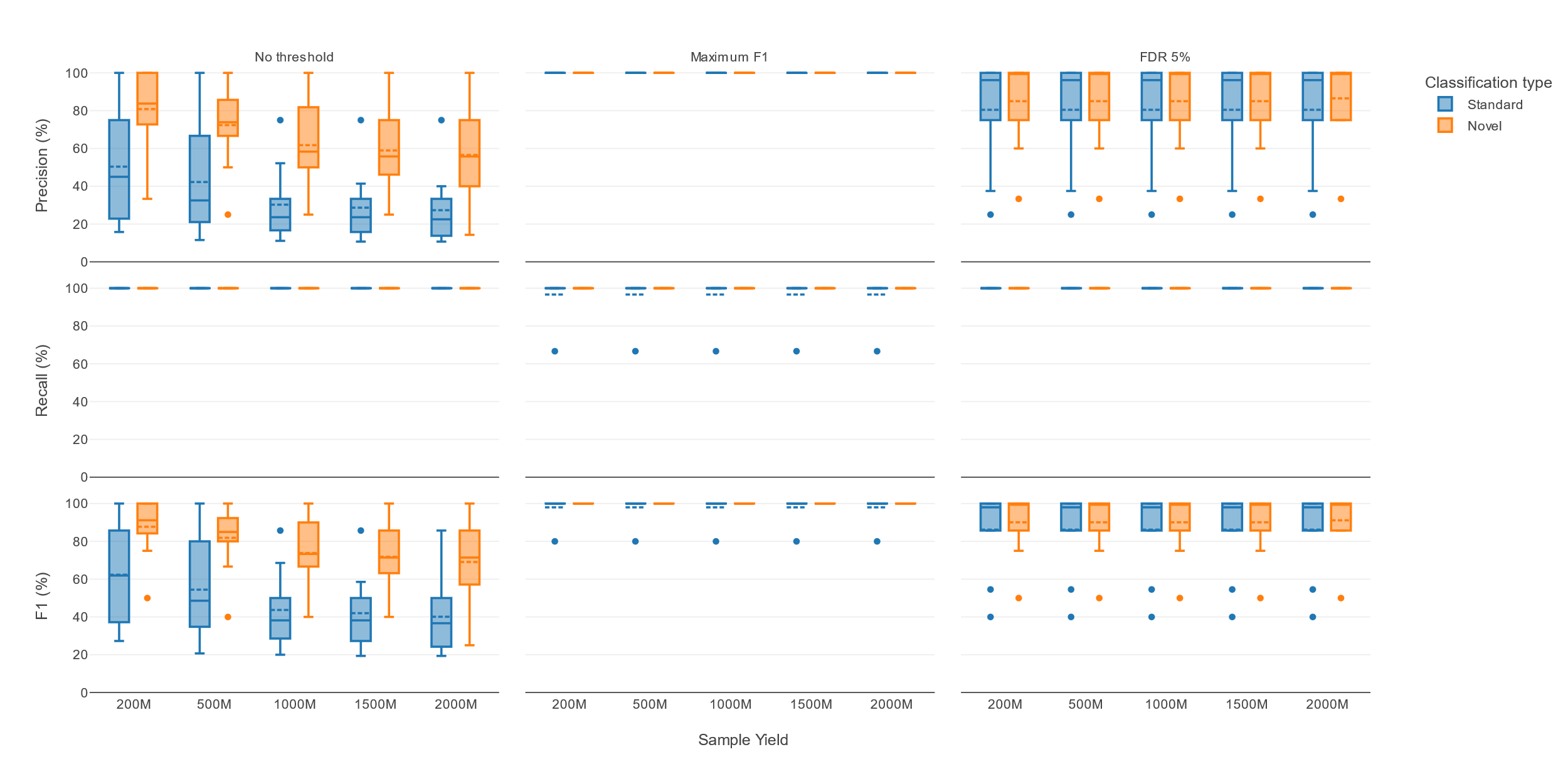


Figure S3: Precision, recall, and F1 scores of the standard and novel approaches applied to the PDMCs. Rows show precision, recall, and F1 scores, while columns represent the three selection strategies: no thresholds applied, maximum F1 score, and FDR5. The x-axis shows the five sample yields, whereas the y-axis shows the respective metric values of the ten probiotic samples expressed as boxplots. Blue and orange boxplots indicate the performance for the standard and novel approaches, respectively, with solid and dotted lines indicating the median and mean of each boxplot. Note that the used thresholds were based on the PDMCs and not the ones from the DMCs. The used filter thresholds are shown in Table S9.

## Figure S4


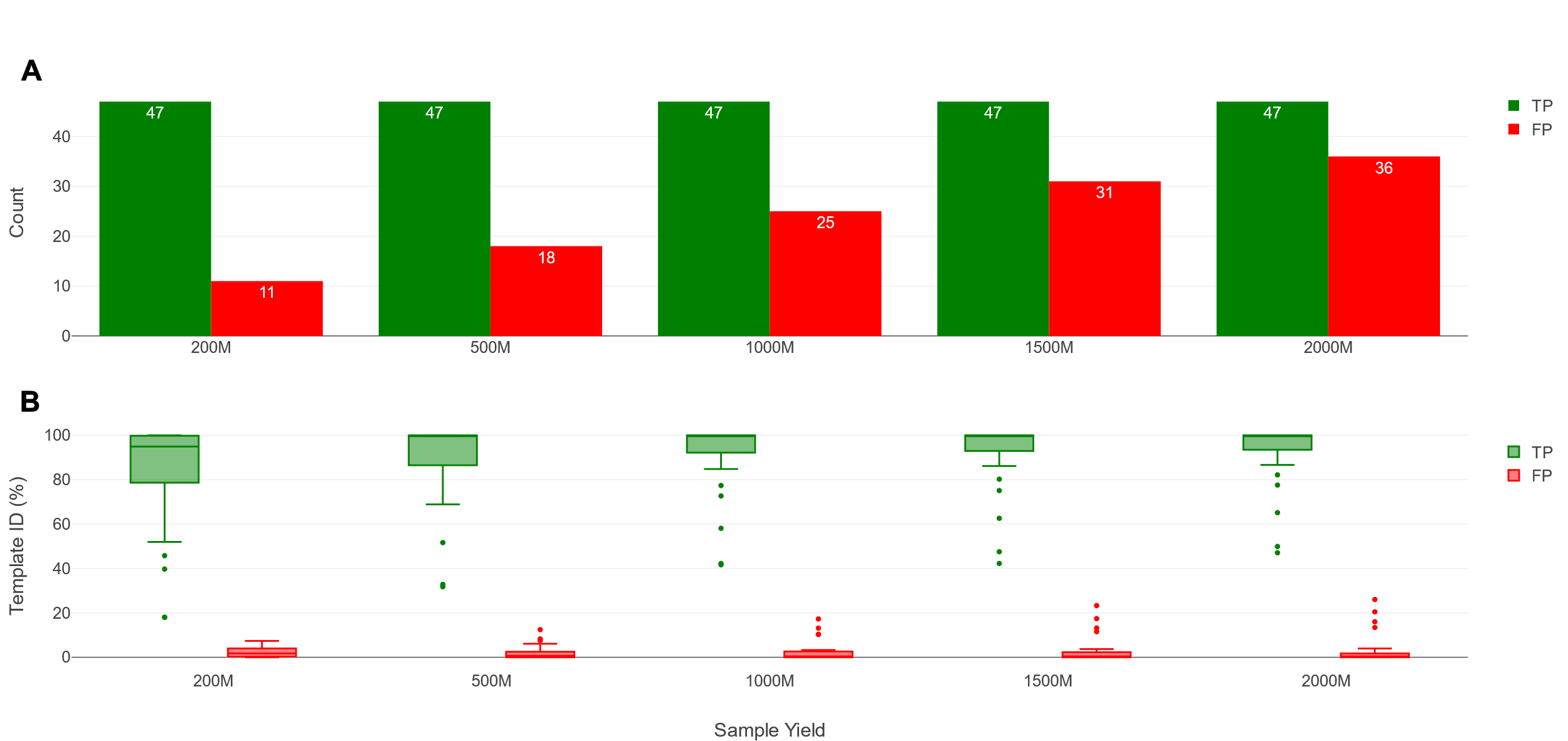


Figure S4: Effects of sequencing yield on TPs, FPs and FNs for the novel approach applied to the probiotic-derived mock communities. (A) Number of TPs, FPs and FNs for all ten probiotic-derived mock communities using the novel approach without filtering. The x-axis shows the five different yields, while the y-axis shows the counts of TPs (green bars), FPs (red bars), and FNs (blue bars). (B) Template IDs of TPs and FPs for all ten probiotic-derived mock communities using the novel approach without filtering. The x-axis shows the five different yields, while the y-axis shows the template ID. Green and red boxplots indicate TPs and FPs, respectively, with a solid line indicating the median. FNs are not shown as they all have a template ID of 0%.

## Figure S5


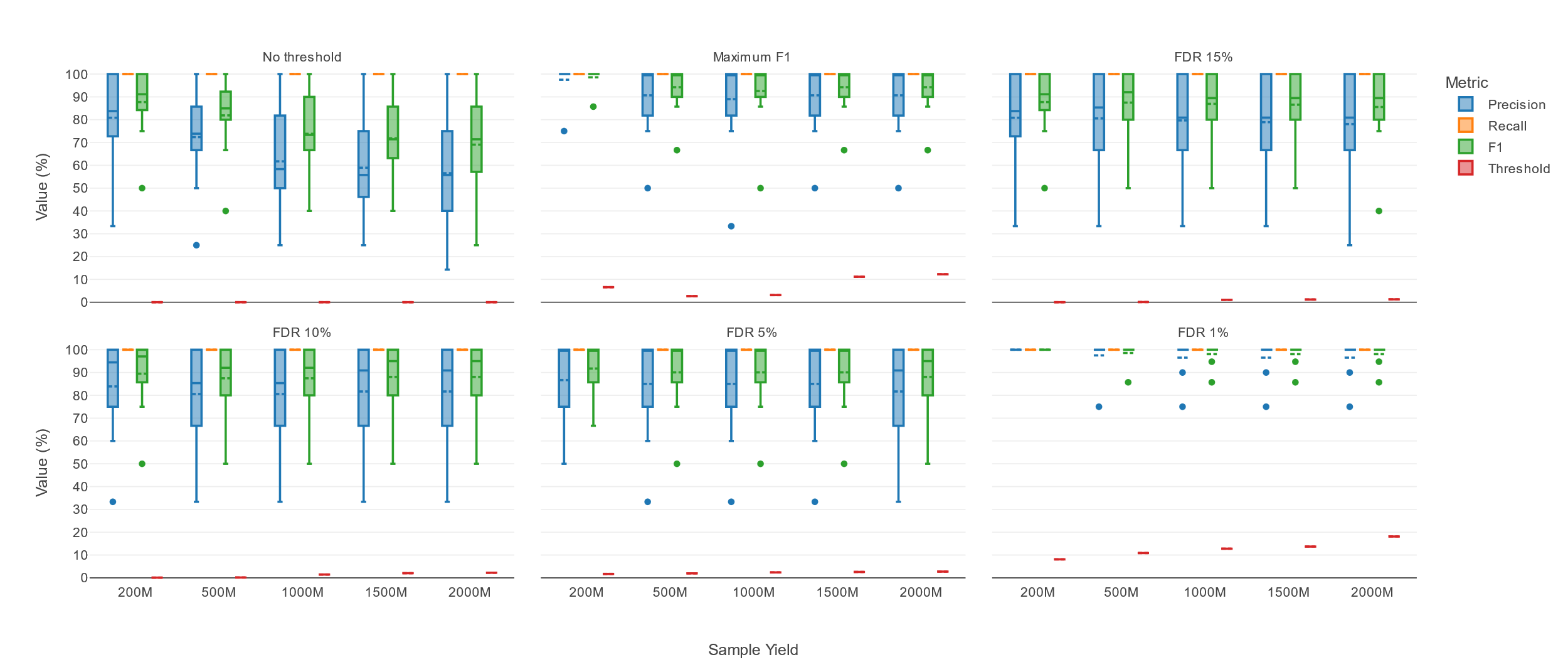


Figure S5: Effects of DMC-derived template ID filtering for different selection strategies across different sequencing yields applied to the probiotic-derived mock communities. Each subplot represents the six different selection strategies. The x-axis represents the different sequencing yields, while the y-axis shows the value (%) of the performance metrics and associated DMC-derived template ID thresholds (see also Figure 4). Blue, orange, green and red boxplots indicate the precision, recall, F1 and template ID threshold of all ten probiotic-derived mock communities.

## Figure S6


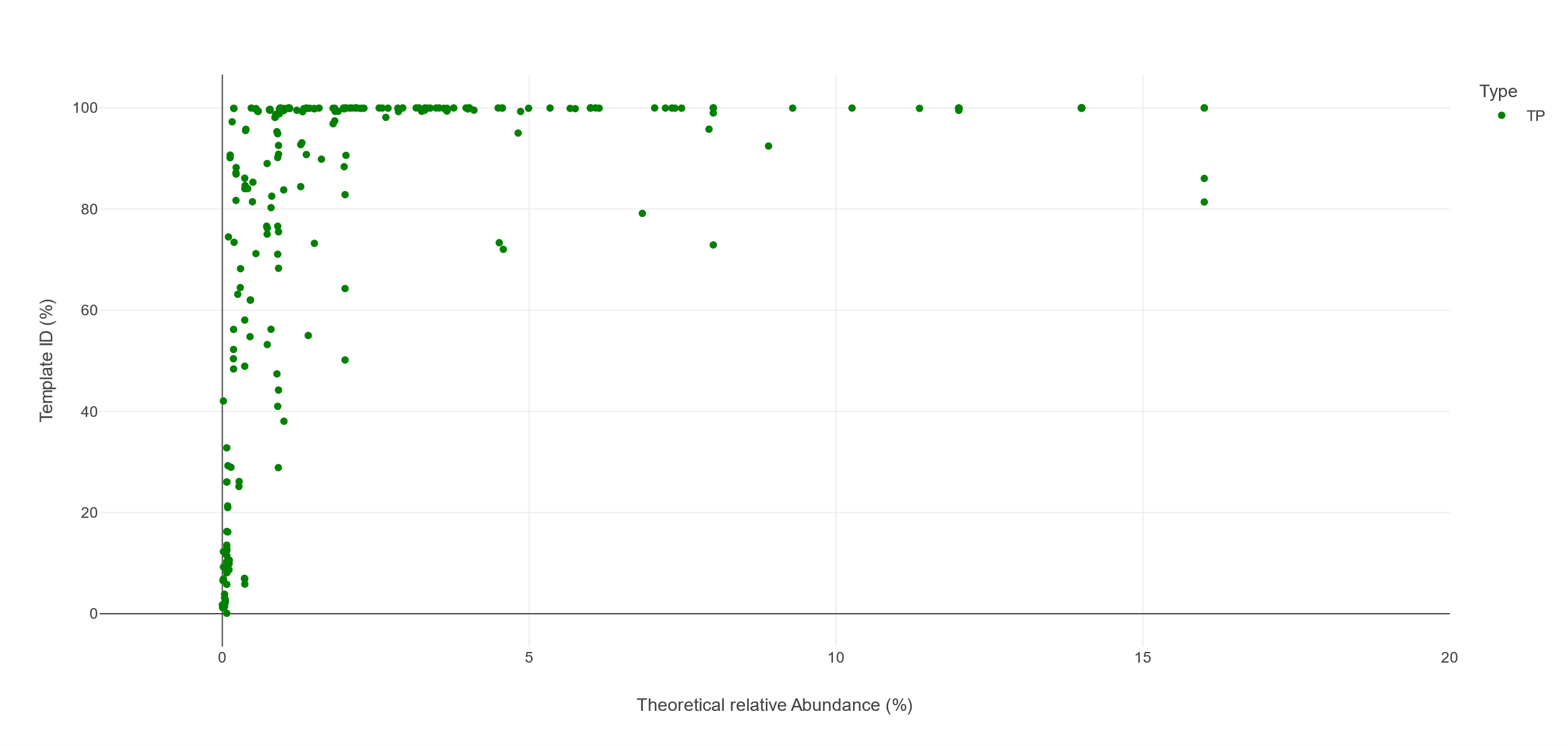


Figure S6: Template ID and theoretical relative abundance of all TPs in the ten DMCs at yield 2000M. The x-axis shows the theoretical relative abundance (%), while the y-axis shows the template ID (%). Green markers represent the TPs.

# References

1. Van Uffelen, A. *et al.* Benchmarking bacterial taxonomic classification using nanopore metagenomics data of several mock communities. *Sci. Data* **11**, (2024).

2. Shen, W. & Ren, H. TaxonKit: A practical and efficient NCBI taxonomy toolkit. *J. Genet. Genomics* **48**, 844–850 (2021).

3. Ciufo, S. *et al.* Using average nucleotide identity to improve taxonomic assignments in prokaryotic genomes at the NCBI. *Int. J. Syst. Evol. Microbiol.* **68**, 2386–2392 (2018).

4. Stoddard, S. F., Smith, B. J., Hein, R., Roller, B. R. K. & Schmidt, T. M. rrnDB: improved tools for interpreting rRNA gene abundance in bacteria and archaea and a new foundation for future development. *Nucleic Acids Res.* **43**, D593–D598 (2014).

5. Bepeyeva, A. *et al.* Encapsulation of Lactobacillus casei into Calcium Pectinate‐Chitosan Beads for Enteric Delivery. *J. Food Sci.* **82**, 2954–2959 (2017).

6. Tejero-Sariñena, S., Barlow, J., Costabile, A., Gibson, G. R. & Rowland, I. Antipathogenic activity of probiotics against Salmonella Typhimurium and Clostridium difficile in anaerobic batch culture systems: Is it due to synergies in probiotic mixtures or the specificity of single strains? *Anaerobe* **24**, 60–65 (2013).

7. Rodenes, A. *et al.* *Reclassification of Probiotic* Lactobacillus Acidophilus *NCIMB 30184 as* Lactobacillus Helveticus *and* Lactobacillus Casei *NCIMB 30185 as* Lacticaseibacillus Paracasei. http://biorxiv.org/lookup/doi/10.1101/2022.10.17.512536 (2022) doi:10.1101/2022.10.17.512536.
